# ETHNICITY AND HEALTH-RELATED QUALITY OF LIFE IN THE POST-STROKE POPULATION: A SYSTEMATIC REVIEW

**DOI:** 10.2340/jrm.v57.41038

**Published:** 2025-01-03

**Authors:** Ying Xing LEE, Cornelie D. ANDELA, Korné JELLEMA, Johannes W. SCHOONES, Thea P. M. VLIET VLIELAND, Henk J. ARWERT

**Affiliations:** 1 Haaglanden Medical Center, Department of Neurology, The Hague; 2 Basalt Rehabilitation Center, The Hague; 3 Directorate of Research Policy, Leiden University Medical Center, Leiden; 4 Department of Orthopaedics, Rehabilitation and Physical Therapy, Leiden University Medical Center, Leiden; 5 Haaglanden Medical Center, Department of Rehabilitation, The Hague, The Netherlands

**Keywords:** stroke, outcome, ethnicity, quality of life, health-related quality of life

## Abstract

**Background/Objective:**

A systematic review was conducted on the association between ethnicity and health-related quality of life in post-stroke populations.

**Methods:**

In February 2024, a comprehensive search was conducted across several databases. Studies were included when they had at least 2 distinct ethnic post-stroke groups for comparison, along with the utilization of validated questionnaires to measure health-related quality of life. Two authors independently screened, selected, and evaluated studies, while 1 author extracted outcome data. When possible, effect sizes were calculated using raw data from included studies.

**Results:**

Eleven studies were included, comprising 12,430 patients. All but 1 study found ethnic disparities in post-stroke health-related quality of life. In 8 studies, patients from minority ethnic groups had lower health-related quality of life after stroke compared with the predominant ethnic group in a country. In 2 studies, the minority group (Asians and non-Hispanic blacks, respectively) showed better outcomes compared with the majority group. In 1 study no differences were observed. In 6 studies the effect size was calculable, and ranged from small to moderate.

**Conclusion:**

Included studies show a large heterogeneity regarding included populations and reported outcomes. Racial/ethnic disparities in stroke patients exist in most studies from different countries. Further studies are needed to investigate the background of these disparities.

## LAY ABSTRACT

Stroke is a growing worldwide problem that can cause long-term disabilities and a negative impact on the quality of life, known as health-related quality of life. Understanding the factors that can influence health-related quality of life is important for improving healthcare services for these patients. One of these possible factors is ethnicity; however, there are limited data available regarding this topic. This article collects, summarizes, and reviews all known data worldwide that address this issue to answer the question of whether ethnicity is associated with health-related quality of life in patients after stroke. This review is one of the first steps to show that ethnic disparities do exist and highlights the importance of further studies to understand the underlying mechanisms of these disparities, with the goal of achieving equality among all groups.

Stroke remains a global health challenge, ranking prominently as a major cause of both mortality and disability worldwide (1). An increase in stroke survivors is expected because of the ageing population and a decline in mortality driven by improvement in stroke care (2, 3). Long-term outcome of stroke varies greatly among patients, but overall stroke is causing long-term disabilities within several domains of health (i.e., 6 months after stroke 30% of patients were unable to walk without some assistance, 46% had cognitive deficits, and 26% were institutionalized in a nursing home) (4), with a significant negative impact on the long-term quality of life and social participation (5–8). Insight into patient characteristics and other factors related to the long-term outcomes of stroke is important for the improvement of post-stroke healthcare services in primary, secondary, and/or tertiary care. A factor that has an impact on the long-term outcomes of stroke is ethnicity (9, 10). Ethnicity, characterized as a sociopolitical construct denoting a group of people with shared common national or cultural traditions, has been implicated in influencing various diseases and its treatment, including acute stroke treatment (11, 12). European studies are underrepresented regarding the relation between ethnicity and outcomes on health-related quality of life (HRQOL) after stroke. Wilkinson et al. found ethnic disparities in end-stage kidney disease at all stages of progression of the disease, its management, and also the long-term quality of life (13). This could also be seen in myocardial infarction by Bansal et al. (14). These disparities may also exist in the post-stroke population. The aim of the current systematic review was to assess and summarize the available data from previous studies regarding the association between ethnicity and HRQOL in the post-stroke population; knowledge regarding this association can support post-stroke healthcare services.

## METHODS

### Search strategy and selection criteria

We performed this systematic review in accordance with the preferred reporting items for systematic reviews and meta-analysis (PRISMA) guidelines (15, 16). The methodology was registered before starting on the PROSPERO online database of systematic reviews in November 2022 (CRD42023355901). The protocol can be accessed from: https://www.crd.york.ac.uk/PROSPERO/display_record.php?RecordID=355901.

We performed a literature search using a search strategy including 3 main terms: “post-stroke population”, “quality of life”, and “ethnicity”. Seven databases were used: PubMed, Embase, Web of Science, Cochrane Library, Emcare, PsycINFO, and Academic Search Premier. The full search strategy is presented in Appendix S1. From the relevant studies, the reference lists were examined to retrieve other eligible studies. Studies were included when they have at least 2 ethnic groups with adults who had experienced stroke, to compare HRQOL (measured with a structured and validated questionnaire) in these groups. Peer-reviewed journal publications regardless of design were included. We excluded studies that were systematic reviews, letters to the editor, meeting abstracts, and protocol descriptions. We also excluded studies if these: (*i*) had no comparison group, i.e., studies only describing outcomes in one ethnic group; (*ii*) had an outcome other than HRQOL; however, if HRQOL was studied as a secondary outcome, the study was included; and (*iii*) had stroke as a part of cardiovascular disease without specification of details (i.e., number of patients with stroke in different ethnic groups), and where stroke data could not be extracted. No limits were placed on the definition of ethnic groups, date of publication, or language of studies.

The electronic search was conducted by 2 researchers (CDA and YXL). The reviewers screened title and abstracts and selected the eligible records based on the inclusion and exclusion criteria. Disagreements regarding the eligibility of the studies for inclusion were resolved by discussion and consensus among the researchers. Data obtained were: baseline characteristics of the population, stroke type and stroke treatment, date of the study, geographic location of the study, the ethnic groups, and outcomes of the valid questionnaires. Information on depression, anxiety, and healthcare usage were reported if available. Due to expected heterogeneity of ethnic groups in studies, subgroups of majority and minority groups were created from included patients in order to support interpretation of the data. The majority group in this systematic review was defined as the predominant ethnic population within the country of the study. In studies including international data, the majority group was defined as the ethnic group with the highest representation of patients. The minority group was defined as the ethnic group(s) without the majority group. For each study, the correction for possible confounders was extracted and described.

### Risk of bias assessment

Study quality was assessed independently by 2 authors (CDA and YXL). The Newcastle-Ottawa Scale for case-control studies was used to assess the study quality (16). Any disagreement was resolved by discussion and consensus. This scale is a checklist with 8t items that consists of 3 quality components: selection, comparability, and outcome. Each item can be scored as a maximum of 1 point, except for the item comparability, which could be scored with a maximum 2 points, with a summed total score ranging from 0 to 9, with lower scores indicating high risk of bias. Previous research categorized the risk of bias score of individual studies into high, moderate, and low risk of bias. Summed scores were grouped into high (0–4), moderate (5–6), and low (7–9) risk of bias. Studies were not excluded based on their risk of bias assessment, considering the low number of included studies.

### Statistical analysis

To compare outcomes of studies using heterogeneous HRQOL measurements, we used Cohen’s d as an indicator for the effect size (ES). Cohen’s d was calculated by dividing the mean differences of the group by the standard deviation (SD), if raw data were provided in included studies (17). Cohen’s d defined measures of small, medium, and large ES as 0.2, 0.5, and 0.8, respectively (17). An ES larger than 0.5 was deemed clinically important.

## RESULTS

### Selection of studies

**Fig. 1:**
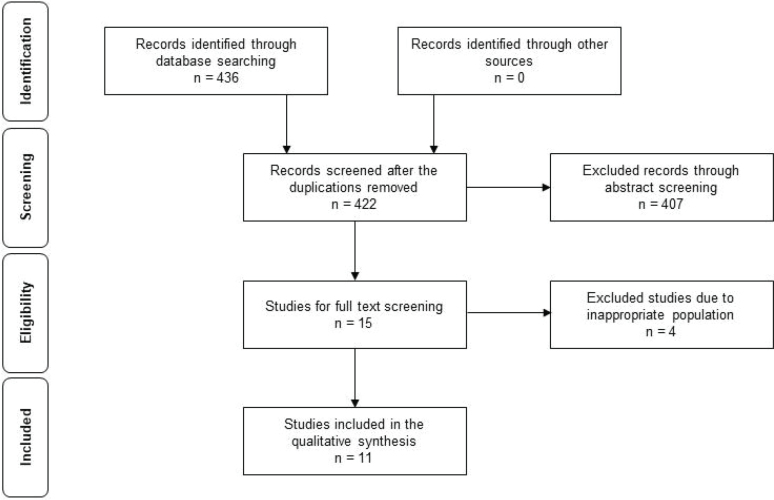
Flow diagram of the selection process of the included studies.

A total of 436 relevant citations were identified through the electronic database search in February 2024 (see Appendix S1 for the detailed search strategy). These studies were screened for eligibility. After title and abstract review, and the removal of duplicate references, 15 full-text articles were retrieved for detailed examination. Among these studies, 4 studies were excluded due to inappropriate population (e.g., patients with diagnoses other than stroke). There were no additional studies identified from the reference lists. Therefore, 11 studies were included in this systematic review (see flow diagram, Fig. 1) (18–28).

### Characteristics of included studies

Table I provides a summary of the characteristics of the included studies. In total, 12,430 patients were included from primary studies, with sample sizes ranging from 107 to 4,016 patients (median of 1,011 patients). Seven studies were retrospective cohort studies and 4 studies were prospective cohort studies. The onset of stroke varied between 1996 and 2019. Two studies used a questionnaire in which patients had to self-report the occurrence of stroke. In these studies, no time of onset of stroke was described (20,21). Three studies exclusively included acute ischaemic stroke patients (17, 19, 23), 2 studies focused solely on spontaneous intracerebral haemorrhage patients (18, 22), 1 study included only spontaneous subarachnoid haemorrhage patients (24) and 3 studies included both ischaemic and haemorrhagic strokes (21, 25, 26).

**Table I:** Study characteristics of included studies

First author, publication year, and country	Study design	Sample size, mean age in years, sex (%)	Ethnicity (%)	Outcome assessment and questionnaire	Follow-up term and loss to follow-up	Outcome HRQOL	ES
Chen, 2020, Australia (patients included in 13 countries, predominantly Asian) (17)	Prospective, originally designed as randomized controlled trial	4,016, mean age 66.1, 37.6% women	Majority group: 67.0% Asian (62.9% Chinese) Minority group: 33.0% non-Asian	3-level version of EQ-5D, by telephone or in person by trained independent researchers	90 days post-randomization, loss to follow-up not described	Non-Asians have poorer HRQOL compared with Asians (predominantly Chinese)	ES calculation not possible
Ellis, 2013, USA (21)	Retrospective cohort study	666, age was dichotomized, 56.2% women, self-reported history of stroke	Majority group: 75.2% non-Hispanic whites Minority groups:12.4% non-Hispanic blacks, 12.5% other	SF-12 (version 2) with MCS and PCS, self-reported	Not clear, patients were included when they had a self-reported history of stroke. Loss to follow-up was not described	Higher PCS in non-Hispanic blacks (minority) compared with non-Hispanic whites or others. No significant differences in MCS	ES on raw data: < 0.2
Griauzde, 2019, USA (22)	Retrospective cohort study (intracerebral haemorrhage stroke)	107, mean age 68.0, 46.7% women	Majority group: 29.9% non-Hispanic whites Minority group: 70.1% Mexican Americans	SSQOL by interviews	90 days post-stroke, loss to follow-up described	No difference by ethnicity in HRQOL outcomes	ES not relevant
Jacobs, 2023, USA (27)	Retrospective cross-sectional study	658, mean age 65.8, 57.8% women	Majority group: 68.8% Whites, 14.9% Blacks, 5.6% Asian/AIAN/other/multiple	HALex score, by interviews	No follow-up	Majority group (Whites) had a significant better HALex value compared with Blacks (18.2% lower scores compared with Whites)	ES calculation not possible
Krishnan, 2017, UK (international data) (18)	Retrospective cohort study	1,011, mean age 66.2, 36.8% women	Majority group: 78.1% Caucasian Minority groups: 14.5% Asians, 5.5% Blacks, < 2% other (which were removed from analyses)	Health utility status derived from EQ-5D and EQ-VAS, self-reported questionnaires	90 days post-stroke, loss to follow-up not described	Majority group (Caucasians) had worse quality of life compared with minority group (Asians)	ES: 0.6
Lee, 2024, the Netherlands (26)	Retrospective cohort study	207, mean age 63.8, 39.6% women	Majority group: 81.6% patients with a European background, 18.4% patients with a non-European background	SF-36 (with MCS and PCS) and EQ-5D self-reported questionnaires	Data collected 2–5 years after diagnosis of stroke	Majority group (patients with a European background) had a significant higher EQ-5D and PCS	ES for PCS: 0.6 ES for EQ-5D: 0.7
Lisabeth, 2022, USA (23)	Prospective population-based, longitudinal study	1332, median age 67, 48.5% women	Majority group: 39.4% non-Hispanic whites Minority group: 60.6% Mexican Americans	Self-report by patient or by proxy using the SSQOL	3, 6 and 12 months post-stroke, loss to follow-up not described	Minority group (Mexican Americans) had worse outcomes compared with the majority group (non-Hispanic whites) at all time points (3, 6, and 12 months)	ES estimated < 0.2
Reeves, 2015, USA (19)	Retrospective cohort study	749 applicable, 290 included, median age 69, 51–54% women	Majority group: 34% non-Hispanic whites Minority group: 66% Mexican Americans	In person or by phone by interviewer using the 12 items SSQOL	90 days post-stroke, loss to follow-up described	Minority group (Mexican Americans) had an overall lower poststroke HRQOL than the majority group (non-Hispanic whites), specifically in the physical domain. No ethnic difference in the psychosocial domain	ES: 0.3
Taufique, 2016, USA (24)	Prospective cohort study (subarachnoid bleeding)	1,181 applicable, 724 included with completed HRQOL outcome, mean age 52, sex not described	Majority group: 51% Whites Minority group: 49% non-Whites (not specified)	In person or by phone by interviewer using the SIP (patient or nearest relative)	12 months post-stroke, loss to follow-up described	Non-white ethnicity (minority group) was a predictor of poor overall and psychosocial HRQOL	ES calculation not possible
Thompson, 2022, New Zealand (25)	Prospective, nationwide, multi-centre observational study	2,379, median age 78, 48.8% women	Majority group: 76.7% European Minority groups: 11.5% Māori, 4.8% Pacific people, 4.8% Asian	Via telephone interviews supplemented by mailed questionnaires using the EQ-5D-3L	3, 6, 12 months post-stroke, loss to follow-up not described	Non-Europeans reported problems with mobility, self-care tasks and pain more often than the majority group (Europeans). The EQ5D-VAS showed no overall differences	ES calculation not possible
Xie, 2006, USA (20)	Retrospective cohort study	1,040, no mean age described, 56.1% women	Majority group: 78% Whites Minority groups: 17.7% Blacks, 4.3% Other	Self-report using the SF-12, EQ-5D index and EQ-VAS	Not clear, patients were included when they had a self-reported history of stroke. Loss to follow-up not described	Lower scores in SF-12 and EQ-5D in blacks compared with the majority group (Whites), and lower scores in EQ-5D in Hispanics compared with non-Hispanics (majority)	ES: < 0.2

*n* = number of patients, MA = Mexican Americans, EQ-5D = Euroqol 5 dimension, EQ-VAS = Euroqol with VAS, SSQoL = Stroke Specific Quality of Life, SF-12 = 12-item Short Form Health Survey, SF-36 = Short Form 36 items, PCS = physical component summary scores, MCS = mental component summary scores, SIP = Sickness Impact Profile, HALex = Health and Activities Limitation index, AIAN = American Indian/Alaska Native, ES = Cohen’s d effect size.

Two studies did not provide a specific definition for stroke (20, 27). Follow-up duration ranged from 90 days to 5 years (duration of follow-up was not reported by 3 studies (20, 21, 27)). The studies originated from 5 countries: United States (*n* = 7), United Kingdom (*n* = 1), the Netherlands (*n* = 1), Australia (*n* = 1) and New Zealand (*n* = 1). The included patients represented diverse ethnic and/or racial backgrounds, including Whites, non-Whites, Blacks, Hispanics, non-Hispanics, Asian (Chinese, other), non-Asian, European, non-European, Maori, and Pacific people. The ethnicity or population group one belongs to was self-reported in 9 studies, 1 study used country of birth (26) and 1 study did not provide this information (17). All studies had HRQOL as outcome. However, 5 different measurements were used. The EQ-5D (Euroqol; with or without VAS) was used 5 times, the SSQoL (Stroke Specific Quality of Life) was used in 3 studies, and the SF-12 (12-item Short Form Health Survey) was used in 2 studies. One study used the SIP (Sickness Impact Profile) and 1 study used the HALex score (Health and Activities Limited index). All studies corrected for possible confounders. The confounders they corrected for were age (17–19, 21, 23, 25–27), sex (17, 19, 20, 22, 23, 25-27), NIHSS (17, 18, 23, 25), functioning pre-stroke using mRS or Barthel score (17, 19, 23, 25, 26), comorbidity/comorbidity index (17, 19, 21, 23), body mass index (19), level of education (19, 23, 24, 26, 27), insurance status (19, 23, 27), income (21, 27), marital status (19, 21, 23, 27), location/geographic region (20, 25, 27), cognitive decline (19, 23, 24), residing in a nursing home prior to stroke (19), household size (27), depression (21, 24), having physical limitations (21), having social limitations (21), reduced daily activities (24), inability to return to work (24), need for assistance with instrumental activities of daily living (21), time to treatment (18), intracerebral haemorrhage volume (18), no intravenous thrombolysis in acute ischaemic stroke (23), and admission Hunt and Hes grade (24).

### Risk of bias

The median risk of bias score across all included studies was 6, with a range of 4–7 (Table II). The majority of studies received a moderate quality rating (*n* = 7; 63.6%), 1 study was rated low quality (9.1%) and 3 studies were rated as good quality (27.3%).

**Table II:** Risk of bias assessment (Newcastle-Ottawa Scale)

Selection					Outcomes				

	Representativeness of exposed cohort	Selection of the non-exposed cohort	Ascertainment of exposure	Demonstration that outcome of interest was not present at start of study	Comparability#	Assessment of outcome	Was follow-up long enough for outcomes to occur	Adequacy of follow up of cohorts	Total
Chen, 2020	*	*	*	-	* *	-	-	*	6
Ellis, 2013	*	*	-	-	* *	-	-	-	4
Griauzde, 2019	*	*	*	-	* -	-	-	*	5
Jacobs, 2023	*	*	*	-	* *	-	-	*	6
Krishnan, 2017	*	*	*	-	* *	-	-	-	5
Lee, 2024	*	*	*	-	* *	-	*	*	7
Lisabeth, 2022	*	*	*	-	* *	-	*	*	7
Reeves, 2015	*	*	*	-	* *	-	-	*	6
Taufique, 2016	*	*	*	-	* *	-	*	*	7
Thompson, 2022	*	*	*	-	* -	-	*	*	6
Xie, 2006	*	*	*	-	* *	-	-	*	6

* = 1 point, - = no point, # = a maximum of 2 stars can be given for Comparability.

### Outcomes

Among the 11 included studies, 8 studies reported that majority groups exhibited better post-stroke HRQOL (17, 19, 20, 23, 24–28), 1 found no differences (22) between ethnic groups, and the other 2 studies reported a better post-stroke HRQOL in the minority group (18, 21). Two studies that included Asians showed better outcomes for Asians than the other ethnic groups they were compared with (17, 18); in 1 of these studies Asians were the minority group (18). Mexican Americans were included in 3 studies (19, 22, 23), 2 of which reported lower HRQOL compared with other groups (19, 23). One study showed a better HRQOL in non-Hispanic blacks as the minority group compared with the majority group (non-Hispanic whites) (21). In the analyses of effect sizes, calculations were possible for 6 out of 11 studies (Table I). For 1 study this could not be calculated due to the absence of significant differences in outcomes between ethnic groups (22), while 4 studies solely provided odds ratios or differences in percentage (17, 24, 25, 27). Among the studies with calculable effect sizes, 2 studies had a moderate effect size between 0.5 and 0.8 (18, 26), while 4 studies had a small effect size (<0.3) (19-21, 23).

## DISCUSSION

This systematic review on the relationship between ethnicity and HRQOL after stroke included 11 studies, in which all studies but 1 (23) showed ethnic disparities in HRQOL after stroke. In 8 studies majority groups had better outcomes than minorities. In studies in which effect sizes could be calculated, most of the studies (4 out of 6 studies) had a small effect size, while 2 studies had a moderate effect size. This suggests that, although statistically significant differences in HRQOL between ethnic groups were observed in most studies, the clinical impact of these differences seems small to medium.

Several underlying mechanisms may contribute to ethnic disparities in post-stroke HRQOL. First, cultural and language barriers may contribute to inequity between ethnic groups. Previous research reported inferior quality of medical care in minority groups despite comparable insurance status, access to healthcare, and severity of conditions (29). Differences in healthcare-seeking behaviour can result from cultural gaps in understanding disease processes and treatment or lack of trust in providers and healthcare systems, which may lead to delay in treatment (29). On the other hand, Asian ethnic groups have a tendency to develop close family bonds, in relation to collectivism as a common feature, in contrast to individualism in other ethnic groups (30, 31). This may attenuate the adverse outcomes after stroke on self-reported questionnaires on HRQOL. A second factor that may contribute to differences in outcome after stroke is access to care facilities, for example due to financial constraints, or differences in the organization of healthcare systems across countries. Differences in health literacy in ethnic groups can also account for differences in health outcomes after stroke (32). Furthermore, genetic factors could be involved. It is widely accepted that genomics have a role in the risk of developing a stroke, directly or by higher incidence of risk factors. To what extent genetic traits influence recovery after stroke in population groups is largely unknown. The understanding of the genetic architecture of ischaemic stroke outcome is still limited (33).

To the best of our knowledge, this is the first systematic review concerning ethnic differences and HRQOL in patients after stroke. A strength of this study is the broad inclusion criteria we used, to ensure inclusion of all studies over time. All studies had their HRQOL measures obtained from blinded researchers. Of the included studies, all except 1 had a moderate or good methodological quality. Also, all studies corrected for possible confounders (i.e., socioeconomic status or post-stroke functional outcome).

However, there are also several limitations to take into account. The major limitation of this systematic review is the heterogeneity in reported outcomes. The included populations differed in most studies, i.e., using different definitions of stroke. Country of origin of the studies also differed, which is important due to differences in healthcare organization and accessibility, therefore making the external validation and generalizability limited. Most studies included data from the United States, which has a different healthcare financing model compared with other countries that contributes to differences in healthcare accessibility (31). In addition, there were several different measurements used for assessing HRQOL. Furthermore, the definition of stroke varied in studies (only haemorrhagic strokes, only subarachnoid haemorrhage, only ischaemic strokes, mixture of both, or not described). Due to these heterogeneous factors, it was impossible to aggregate data to support conclusions. Nevertheless, subgroups were formed into majority and minority groups in order to support interpretation of the data. Last, follow-up time was not described in 3 studies and only 4 studies had a follow-up time longer than 90 days. This might be relevant, as it was reported that recovery of stroke survivors or adaptation of their level of disability may increase HRQOL (34). Future studies would benefit from longer follow-up (> 90 days).

In conclusion, racial or ethnic disparities in stroke patients have a small but distinct effect on HRQOL in different countries. Patients from minority ethnic groups, except Asians, reported a lower HRQOL after stroke, compared with the predominant ethnic group in a country. More insight into the underlying mechanisms of racial or ethnic HRQOL disparities will be valuable for the development of patient care pathways targeting high-priority groups aiming to achieve equal HRQOL outcomes in patients after stroke.

## Supplementary Material



## References

[1] Feigin VL, Stark BA, Johnson CO, Roth GA, Bisignano C, Abady GG, et al. Global, regional, and national burden of stroke and its risk factors, 1990–2019: a systematic analysis for the Global Burden of Disease Study 2019. Lancet Neurol 2021; 20: 1–26. 10.1016/S1474-4422(21)00252-0 34487721 PMC8443449

[2] Béjot Y, Blanc C, Delpont B, Thouant P, Chazalon C, Daumas A, et al. Increasing early ambulation disability in spontaneous intracerebral hemorrhage survivors. Neurology 2018; 90: e2017–2024. 10.1212/WNL.0000000000005633 29728525

[3] Seminog OO, Scarborough P, Wright FL, Rayner M, Goldacre MJ. Determinants of the decline in mortality from acute stroke in England: linked national database study of 795 869 adults. BMJ 2019; 365. 10.1136/bmj.l1778 PMC6529851 31122927

[4] Go AS, Mozaffarian D, Roger VL, Benjamin EJ, Berry JD, Borden WB, et al. Heart disease and stroke statistics – 2013 update: a report from the American Heart Association. Circulation 2013; 127: e6–e245. 23239837 10.1161/CIR.0b013e31828124ad PMC5408511

[5] Ovbiagele B, Goldstein LB, Higashida RT, Howard VJ, Johnston SC, Khavjou OA, et al. Forecasting the future of stroke in the United States: a policy statement from the American Heart Association and American Stroke Association. Stroke 2013; 44: 2361–2375. 10.1161/STR.0b013e31829734f2 23697546

[6] Arwert HJ, Groeneveld IF, Vliet Vlieland TPM, Meesters JJL. Health care use and its associated factors 5–8 years after stroke. J Stroke Cerebrovasc Dis 2019; 28: 1–6. 10.1016/j.jstrokecerebrovasdis.2019.104333 31455556

[7] Ayerbe L, Ayis SA, Crichton S, Wolfe CDA, Rudd AG. Natural history, predictors and associated outcomes of anxiety up to 10 years after stroke: the South London Stroke Register. Age Ageing 2014; 43: 542–547. 10.1093/ageing/aft208 24375225

[8] Groeneveld IF, Arwert HJ, Goossens PH, Vliet Vlieland TPM. The Longer-term Unmet Needs After Stroke Questionnaire: cross-cultural adaptation, reliability, and concurrent validity in a Dutch population. J Stroke Cerebrovasc Dis 2018; 27: 267–275. 10.1016/j.jstrokecerebrovasdis.2017.08.043 28967592

[9] Tsalta-Mladenov M, Andonova S. Health-related quality of life after ischemic stroke: impact of sociodemographic and clinical factors. Neurol Res 2021; 43: 553–561. 10.1080/01616412.2021.1893563 33637026

[10] Ellis C, Hyacinth HI, Beckett J, Feng W, Chimowitz M, Ovbiagele B, et al. Racial/ethnic differences in poststroke rehabilitation outcomes. Stroke Res Treat 2014; 2014: 950746. 10.1155/2014/950746 25028619 PMC4084586

[11] Ford ME, Kelly PA. Conceptualizing and categorizing race and ethnicity in health services research. Health Serv Res 2005; 40: 1658–1675. 10.1111/j.1475-6773.2005.00449.x 16179001 PMC1361221

[12] Green TL, Singh P, King-Shier K. The impact of ethnic/racial status on access to care and outcomes after stroke: a narrative systematic review. J Vasc Nurs 2019; 37: 199–212. 10.1016/j.jvn.2019.07.002 31727312

[13] Wilkinson E, Brettle A, Waqar M, Randhawa G. Inequalities and outcomes: end stage kidney disease in ethnic minorities. BMC Nephrol 2019; 20: 1–12. 10.1186/s12882-019-1410-2 31242862 PMC6595597

[14] Bansal M, Mehta A, Balakrishna AM, Poppas A, Abbott JD, Vallabhajosyula S. et al. Race, ethnicity, and gender disparities in acute myocardial infarction. Critical Care Clinics 2024; 40: 685–707. 10.1016/j.ccc.2024.05.005 39218481

[15] Hutton B, Salanti G, Caldwell DM, Chaimani A, Schmid CH, Cameron C, et al. The PRISMA extension statement for reporting of systematic reviews incorporating network meta-analyses of health care interventions: checklist and explanations. Ann Intern Med 2015; 162: 777–784. 10.7326/M14-2385 26030634

[16] Moher D, Liberati A, Tetzlaff J, Altman DG. Preferred reporting items for systematic reviews and meta-analyses: the PRISMA statement. BMJ 2009; 339: 332–336. 10.1136/bmj.b2535 PMC3090117 21603045

[17] Cohen J. Statistical power analysis for the behaviorial sciences. 2nd ed. Mahwah, NJ: Lawrence Erlbaum Associates, 1988.

[18] Chen X, Wang X, Delcourt C, Li J, Arima H, Hackett ML, et al. Ethnicity and other determinants of quality of functional outcome in acute ischemic stroke: the ENCHANTED trial. Stroke 2020;51: 588–593. 10.1161/STROKEAHA.119.027639 31822251

[19] Krishnan K, Beishon L, Berge E, Christensen H, Dineen RA, Ozturk S, et al. Relationship between race and outcome in Asian, Black, and Caucasian patients with spontaneous intracerebral hemorrhage: data from the Virtual International Stroke Trials Archive and Efficacy of Nitric Oxide in Stroke trial. Int J Stroke 2018; 13: 362–373. 10.1177/1747493017744463 29165060

[20] Reeves SL, Brown DL, Baek J, Wing JJ, Morgenstern LB, Lisabeth LD. Ethnic differences in poststroke quality of life in the brain attack surveillance in Corpus Christi (BASIC) project. Stroke 2015; 46: 2896–2901. 10.1161/STROKEAHA.115.010328 26286542 PMC4589482

[21] Xie J, Wu EQ, Zheng ZJ, Croft JB, Greenlund KJ, Mensah GA, et al. Impact of stroke on health-related quality of life in the noninstitutionalized population in the United States. Stroke 2006; 37: 2567–2572. 10.1161/01.STR.0000240506.34616.10 16946158

[22] Ellis C, Grubaugh AL, Egede LE. Factors associated with SF-12 physical and mental health quality of life scores in adults with stroke. J Stroke Cerebrovasc Dis 2013; 22: 309–317. 10.1016/j.jstrokecerebrovasdis.2011.09.007 22005038

[23] Griauzde J, Lisabeth LD, Li C, Sanchez BN, Case E, Garcia NM, et al. A population-based study of intracerebral hemorrhage survivors’ outcomes. J Stroke Cerebrovasc Dis 2020; 28: 49–55. 10.1016/j.jstrokecerebrovasdis.2018.09.005 PMC6289885 30274873

[24] Lisabeth LD, Brown DL, Dong L, Zahuranec DB, Kwicklis M, Shi X, et al. Outcomes in the year after first-ever ischemic stroke in a bi-ethnic population. Ann Neurol 2023; 93: 348–356. 10.1002/ana.26513 36134521 PMC9892337

[25] Taufique Z, May T, Meyers E, Falo C, Mayer SA, Agarwal S, et al. Predictors of poor quality of life 1 year after subarachnoid hemorrhage. Neurosurgery 2016; 78: 256–263. 10.1227/NEU.0000000000001042 26421590

[26] Thompson SG, Barber PA, Gommans JH, Cadilhac DA, Davis A, Fink JN, et al. The impact of ethnicity on stroke care access and patient outcomes: a New Zealand nationwide observational study. Lancet Reg Heal – West Pacific 2022; 20: 100358. 10.1016/j.lanwpc.2021.100358 PMC8743211 35036976

[27] Lee YX, Auwerda ST, Jellema K, Vliet Vlieland TPM, Arwert HJ. Ethnic disparities in long-term outcomes and health care usage after stroke in the Netherlands. Disabil Health J 2024; 17: 101582. 10.1016/j.dhjo.2024.101582 38246799

[28] Jacobs MM, Evans E, Ellis C Jr. Disparities in health-related quality of life among adults with ischemic heart disease, stroke, and both conditions: a cross-sectional study. Heart Mind 2023; 7: 171–179. 10.4103/hm.HM-D-23-00025

[29] Padela AI, Punekar IRA. Emergency medical practice: advancing cultural competence and reducing health care disparities. Acad Emerg Med 2009; 16: 69–75. 10.1111/j.1553-2712.2008.00305.x 19055674

[30] Barrett M, Chu A, Chen J, Lam KY, Portenoy R, Dhingra L. Quality of life in community-dwelling Chinese American patients with cancer pain. J Immigr Minor Heal 2017; 19: 1442–1448. 10.1007/s10903-016-0392-4 26993113

[31] Hook JN, Worthington EL, Utsey SO. Collectivism, forgiveness, and social harmony. Counseling Psychologist 2008; 37: 821–847. 10.1177/0011000008326546

[32] Mahajan S, Caraballo C, Lu Y, Valero-Elizondo J, Massey D, Annapureddy AR, et al. Trends in differences in health status and health care access and affordability by race and ethnicity in the United States, 1999–2018. JAMA 2021; 326: 637–648. 10.1001/jama.2021.9907 34402830 PMC8371573

[33] Balkaya M, Cho S. Genetics of stroke recovery: BDNF val66met polymorphism in stroke recovery and its interaction with aging. Neurobiol Dis 2019; 126: 36–46. 10.1016/j.nbd.2018.08.009 30118755 PMC7653673

[34] Barbosa PM, Ferreira LN, Cruz VT, Silva A, Szrek H. Healthcare, clinical factors and rehabilitation predicting quality of life in first-time stroke patients: a 12-month longitudinal study. J Stroke Cerebrovasc Dis 2022; 31: 106300. 10.1016/j.jstrokecerebrovasdis.2021.106300 35081506

