# Supplementary material for: ETHNICITY AND HEALTH-RELATED QUALITY OF LIFE IN THE POST-STROKE POPULATION: A SYSTEMATIC REVIEW
Source: J Rehabil Med. 2025 Jan 3;57:41038. doi: 10.2340/jrm.v57.41038 (PMC11681143; doi:10.2340/jrm.v57.41038)
Supplement: Supplementary file 1 [file JRM-57-41038-s1.pdf]

Supplementary material has been published as submitted. It has not been copyedited, or typeset by Journal of Rehabilitation Medicine

Databases

PubMed

(("Stroke/ethnology"[mesh] OR "Brain ischemia/ethnology"[mesh] OR "Cerebral Hemorrhage/ethnology"[mesh] OR "Ischemic Attack, Transient/ethnology"[mesh] OR (("Stroke"[mesh] OR "Stroke"[tiab] OR "strokes"[tiab] OR "CVA"[tiab] OR "CVAs"[tiab] OR "Cerebrovascular Accident"[tiab] OR "Cerebrovascular Accidents"[tiab] OR "Brain Attack"[tiab] OR "Brain Attacks"[tiab] OR Brain Infarct*[tiab] OR "Brain Infarction"[mesh] OR "Cerebrovascular Apoplexy"[tiab] OR "Apoplexy"[tiab] OR "Brain Vascular Accident"[tiab] OR "Brain Vascular Accidents"[tiab] OR "Cerebrovascular Stroke"[tiab] OR "Cerebrovascular Strokes"[tiab] OR "Cerebral Stroke"[tiab] OR "Cerebral Strokes"[tiab] OR "Acute Stroke"[tiab] OR "Acute Strokes"[tiab] OR "Acute Cerebrovascular Accident"[tiab] OR "Acute Cerebrovascular Accidents"[tiab] OR "Cerebrovascular Event"[tiab] OR "Cerebrovascular Events"[tiab] OR "Cerebrovascular Attack"[tiab] OR "Cerebrovascular Attacks"[tiab] OR "poststroke"[tiab] OR "Cerebral Infarction"[mesh] OR Cerebral infarct*[tiab] OR "Brain ischemia"[mesh] OR "Brain ischemia"[tiab] OR "Brain ischaemia"[tiab] OR "Cerebral Ischemia"[tiab] OR "Cerebral Ischaemia"[tiab] OR "Cerebral Hemorrhage"[mesh] OR Cerebral Hemorrhag*[tiab] OR Cerebral Haemorrhag*[tiab] OR Intracerebral Hemorrhag*[tiab] OR Brain Hemorrhag*[tiab] OR Brain Haemorrhag*[tiab] OR Intracerebral Haemorrhag*[tiab] OR "Neurologic Event"[tiab] OR "Neurologic Events"[tiab] OR "Ischemic Attack, Transient"[mesh] OR "transient ischemic attack"[tiab] OR "transient ischemic attack"[tiab] OR "transient ischaemic attack"[tiab] OR "transient ischaemic attacks"[tiab] OR "TIA"[tiab] OR "TIAs"[tiab] OR "transient brainstem ischemia"[tiab] OR "transient brainstem ischaemia"[tiab] OR "transient brain stem ischemia"[tiab] OR "transient brain ischemia"[tiab] OR "transient brain ischaemia"[tiab] OR "transient cerebral ischemia"[tiab] OR "transient cerebral ischaemia"[tiab] OR "transient cerebral ischemic"[tiab] OR "transient cerebral ischaemic"[tiab] OR ("transient"[tiab] AND ("brain"[tiab] OR "cerebral"[tiab]) AND (ischemi*[tiab] OR ischaem*[tiab]))) AND ("Ethnicity"[Mesh] OR "Ethnology"[Mesh] OR "Population Groups"[mesh] OR "Population Groups"[tiab] OR "Population Group"[tiab] OR "ethnic"[tiab] OR "ethnicity"[tiab] OR "ethnic*"[tiab] OR "ethnology"[tiab] OR "ethnol*"[tiab] OR "Indians"[tiab] OR "Asians"[tiab] OR "Asian Americans"[tiab] OR "Blacks"[tiab] OR "African Americans"[tiab] OR "Whites"[tiab] OR "Hispanics"[tiab] OR "Latino"[tiab] OR "Mexican Americans"[tiab] OR "Indigenous"[tiab] OR "ancestry"[tiab] OR "Emigrants and Immigrants"[Mesh] OR "Emigrants"[tiab] OR "Emigrant"[tiab] OR "emigrat*"[tiab] OR "Immigrants"[tiab] OR "Immigrant"[tiab] OR "immigrat*"[tiab] OR "Refugees"[Mesh] OR "Refugees"[tiab] OR "Refugee"[tiab] OR "Asylum Seekers"[tiab] OR "Asylum Seeker"[tiab] OR "Displaced Persons"[tiab] OR "Displaced Person"[tiab] OR "sociodemographic factor"[tiab] OR "sociodemographic factors"[tiab] OR "socio demographic factor"[tiab] OR "socio demographic factors"[tiab]))) AND ("Quality of Life"[mesh] OR "Quality of Life"[tiab] OR "QoL"[tiab] OR "HRQoL"[tiab] OR "life quality"[tiab] OR "Sickness Impact Profile"[Mesh] OR "Sickness Impact Profile"[tiab]))

Embase

(((exp *"cerebrovascular accident"/ OR "Stroke".ti OR "strokes".ti OR "CVA".ti OR "CVAs".ti OR "Cerebrovascular Accident".ti OR "Cerebrovascular Accidents".ti OR "Brain Attack".ti OR "Brain Attacks".ti OR "Brain Infarction".ti OR "Brain Infarctions".ti OR "Brain Infarct*".ti OR exp *"Brain Infarction"/ OR "Cerebrovascular Apoplexy".ti OR "Apoplexy".ti OR "Brain Vascular Accident".ti OR "Brain Vascular Accidents".ti OR "Cerebrovascular Stroke".ti OR "Cerebrovascular Strokes".ti OR "Cerebral Stroke".ti OR "Cerebral Strokes".ti OR "Acute Stroke".ti OR "Acute Strokes".ti OR "Acute Cerebrovascular Accident".ti OR "Acute Cerebrovascular Accidents".ti OR "Cerebrovascular Event".ti OR "Cerebrovascular Events".ti OR "Cerebrovascular Attack".ti OR "Cerebrovascular Attacks".ti OR "poststroke".ti OR "Cerebral infarction".ti OR "Cerebral infarctions".ti OR "Cerebral infarct*".ti OR exp *"Brain ischemia"/ OR "Brain ischemia".ti OR "Brain ischaemia".ti OR "Cerebral Ischemia".ti OR "Cerebral Ischaemia".ti OR exp *"Brain Hemorrhage"/ OR Cerebral Hemorrhag*.ti OR Cerebral Haemorrhag*.ti OR Intracerebral Hemorrhag*.ti OR Brain Hemorrhag*.ti OR Brain Haemorrhag*.ti OR Intracerebral Haemorrhag*.ti OR "Neurologic Event".ti OR "Neurologic Events".ti OR exp *"Transient Ischemic Attack"/ OR "transient ischemic attack".ti OR "transient ischemic attack".ti OR "transient ischaemic attack".ti OR "transient ischaemic attacks".ti OR "TIA".ti OR "TIAs".ti OR "transient brainstem ischemia".ti OR "transient brainstem ischaemia".ti OR "transient brain stem ischemia".ti OR "transient brain ischemia".ti OR "transient brain ischaemia".ti OR "transient cerebral ischemia".ti OR "transient cerebral ischaemia".ti OR "transient cerebral ischemic".ti OR "transient cerebral ischaemic".ti OR ("transient".ti AND ("brain".ti OR "cerebral".ti) AND (ischemi*.ti OR ischaem*.ti))) AND (exp *"Ethnicity"/ OR exp *"Ethnology"/ OR exp *"ethnic or racial aspects"/ OR exp *"Ethnic Group"/ OR exp *"Population Group"/ OR "Population Groups".ti,ab OR "Population Group".ti,ab OR "ethnic".ti,ab OR "ethnicity".ti,ab OR "ethnic*".ti,ab OR "ethnology".ti,ab OR "ethnol*".ti,ab OR "Indians".ti,ab OR "Asians".ti,ab OR "Asian Americans".ti,ab OR "Blacks".ti,ab OR "African Americans".ti,ab OR "Whites".ti,ab OR "Hispanics".ti,ab OR "Latino".ti,ab OR "Mexican Americans".ti,ab OR "Indigenous".ti,ab OR "ancestry".ti,ab OR exp *"migrant"/ OR exp *"undocumented immigrant"/ OR "Emigrants".ti,ab OR "Emigrant".ti,ab OR "emigrat*".ti,ab OR "Immigrants".ti,ab OR "Immigrant".ti,ab OR "immigrat*".ti,ab OR exp *"Refugee"/ OR "Refugees".ti,ab OR "Refugee".ti,ab OR "Asylum Seekers".ti,ab OR "Asylum Seeker".ti,ab OR "Displaced Persons".ti,ab OR "Displaced Person".ti,ab OR "sociodemographic factor".ti,ab OR "sociodemographic factors".ti,ab OR "socio demographic factor".ti,ab OR "socio demographic factors".ti,ab) AND (exp *"Quality of Life"/ OR "Quality of Life".ti,ab OR "QoL".ti,ab OR "HRQoL".ti,ab OR "life quality".ti,ab OR exp *"Sickness Impact Profile"/ OR "Sickness Impact Profile".ti,ab)) **OR** ((exp *"cerebrovascular accident"/ OR "Stroke".ti,ab OR "strokes".ti,ab OR "CVA".ti,ab OR "CVAs".ti,ab OR "Cerebrovascular Accident".ti,ab OR "Cerebrovascular Accidents".ti,ab OR "Brain Attack".ti,ab OR "Brain Attacks".ti,ab OR "Brain Infarction".ti,ab OR "Brain Infarctions".ti,ab OR "Brain Infarct*".ti,ab OR exp *"Brain Infarction"/ OR "Cerebrovascular Apoplexy".ti,ab OR "Apoplexy".ti,ab OR "Brain Vascular Accident".ti,ab OR "Brain Vascular Accidents".ti,ab OR "Cerebrovascular Stroke".ti,ab OR "Cerebrovascular Strokes".ti,ab OR "Cerebral Stroke".ti,ab OR "Cerebral Strokes".ti,ab OR "Acute Stroke".ti,ab OR "Acute Strokes".ti,ab OR "Acute Cerebrovascular Accident".ti,ab OR "Acute Cerebrovascular Accidents".ti,ab OR "Cerebrovascular Event".ti,ab OR "Cerebrovascular Events".ti,ab OR "Cerebrovascular Attack".ti,ab OR "Cerebrovascular Attacks".ti,ab OR "poststroke".ti,ab OR "Cerebral infarction".ti,ab OR "Cerebral infarctions".ti,ab OR "Cerebral infarct*".ti,ab OR exp *"Brain ischemia"/ OR "Brain ischemia".ti,ab OR "Brain ischaemia".ti,ab OR "Cerebral Ischemia".ti,ab OR "Cerebral Ischaemia".ti,ab OR exp *"Brain Hemorrhage"/ OR Cerebral Hemorrhag*.ti,ab OR Cerebral Haemorrhag*.ti,ab OR Intracerebral Hemorrhag*.ti,ab OR Brain Hemorrhag*.ti,ab OR Brain Haemorrhag*.ti,ab OR Intracerebral Haemorrhag*.ti,ab OR "Neurologic Event".ti,ab OR "Neurologic Events".ti,ab OR exp *"Transient Ischemic Attack"/ OR "transient ischemic attack".ti,ab OR "transient ischemic attack".ti,ab OR "transient ischaemic attack".ti,ab OR "transient

ischaemic attacks".ti,ab OR "TIA".ti,ab OR "TIAs".ti,ab OR "transient brainstem ischemia".ti,ab OR "transient brainstem ischaemia".ti,ab OR "transient brain stem ischemia".ti,ab OR "transient brain ischemia".ti,ab OR "transient brain ischaemia".ti,ab OR "transient cerebral ischemia".ti,ab OR "transient cerebral ischaemia".ti,ab OR "transient cerebral ischemic".ti,ab OR "transient cerebral ischaemic".ti,ab OR ("transient".ti,ab AND ("brain".ti,ab OR "cerebral".ti,ab) AND (ischemi*.ti,ab OR ischaem*.ti,ab))) AND (exp *"Ethnicity"/ OR exp *"Ethnology"/ OR exp *"ethnic or racial aspects"/ OR exp *"Ethnic Group"/ OR exp *"Population Group"/ OR "Population Groups".ti OR "Population Group".ti OR "ethnic".ti OR "ethnicity".ti OR "ethnic*".ti OR "ethnology".ti OR "ethnol*".ti OR "Indians".ti OR "Asians".ti OR "Asian Americans".ti OR "Blacks".ti OR "African Americans".ti OR "Whites".ti OR "Hispanics".ti OR "Latino".ti OR "Mexican Americans".ti OR "Indigenous".ti OR "ancestry".ti OR exp *"migrant"/ OR exp *"undocumented immigrant"/ OR "Emigrants".ti OR "Emigrant".ti OR "emigrat*".ti OR "Immigrants".ti OR "Immigrant".ti OR "immigrat*".ti OR exp *"Refugee"/ OR "Refugees".ti OR "Refugee".ti OR "Asylum Seekers".ti OR "Asylum Seeker".ti OR "Displaced Persons".ti OR "Displaced Person".ti OR "sociodemographic factor".ti OR "sociodemographic factors".ti OR "socio demographic factor".ti OR "socio demographic factors".ti) AND (exp *"Quality of Life"/ OR "Quality of Life".ti,ab OR "QoL".ti,ab OR "HRQoL".ti,ab OR "life quality".ti,ab OR exp *"Sickness Impact Profile"/ OR "Sickness Impact Profile".ti,ab))) NOT (conference review or conference abstract).pt

Web of Science

((TI=("cerebrovascular accident" OR "Stroke" OR "strokes" OR "CVA" OR "CVAs" OR "Cerebrovascular Accident" OR "Cerebrovascular Accidents" OR "Brain Attack" OR "Brain Attacks" OR "Brain Infarction" OR "Brain Infarctions" OR "Brain Infarct*" OR "Brain Infarction" OR "Cerebrovascular Apoplexy" OR "Apoplexy" OR "Brain Vascular Accident" OR "Brain Vascular Accidents" OR "Cerebrovascular Stroke" OR "Cerebrovascular Strokes" OR "Cerebral Stroke" OR "Cerebral Strokes" OR "Acute Stroke" OR "Acute Strokes" OR "Acute Cerebrovascular Accident" OR "Acute Cerebrovascular Accidents" OR "Cerebrovascular Event" OR "Cerebrovascular Events" OR "Cerebrovascular Attack" OR "Cerebrovascular Attacks" OR "poststroke" OR "Cerebral infarction" OR "Cerebral infarctions" OR "Cerebral infarct*" OR "Brain ischemia" OR "Brain ischemia" OR "Brain ischaemia" OR "Cerebral Ischemia" OR "Cerebral Ischaemia" OR "Brain Hemorrhage" OR Cerebral Hemorrhag* OR Cerebral Haemorrhag* OR Intracerebral Hemorrhag* OR Brain Hemorrhag* OR Brain Haemorrhag* OR Intracerebral Haemorrhag* OR "Neurologic Event" OR "Neurologic Events" OR "Transient Ischemic Attack" OR "transient ischemic attack" OR "transient ischemic attack" OR "transient ischaemic attack" OR "transient ischaemic attacks" OR "TIA" OR "TIAs" OR "transient brainstem ischemia" OR "transient brainstem ischaemia" OR "transient brain stem ischemia" OR "transient brain ischemia" OR "transient brain ischaemia" OR "transient cerebral ischemia" OR "transient cerebral ischaemia" OR "transient cerebral ischemic" OR "transient cerebral ischaemic" OR ("transient" AND ("brain" OR "cerebral") AND (ischemi* OR ischaem*))) AND (TI=("Ethnicity" OR "Ethnology" OR "ethnic or racial aspects" OR "Ethnic Group" OR "Population Group" OR "Population Groups" OR "Population Group" OR "ethnic" OR "ethnicity" OR "ethnic*" OR "ethnology" OR "ethnol*" OR "Indians" OR "Asians" OR "Asian Americans" OR "Blacks" OR "African Americans" OR "Whites" OR "Hispanics" OR "Latino" OR "Mexican Americans" OR "Indigenous" OR "ancestry" OR "migrant" OR "undocumented immigrant" OR "Emigrants" OR "Emigrant" OR "emigrat*" OR "Immigrants" OR "Immigrant" OR "immigrat*" OR "Refugee" OR "Refugees" OR "Refugee" OR "Asylum Seekers" OR "Asylum Seeker" OR "Displaced Persons" OR "Displaced Person" OR "sociodemographic factor" OR "sociodemographic factors" OR "socio demographic factor" OR "socio demographic factors") OR AK=("Ethnicity" OR "Ethnology" OR "ethnic or racial aspects" OR "Ethnic Group" OR "Population Group" OR "Population Groups" OR "Population Group" OR "ethnic" OR "ethnicity" OR "ethnic*" OR "ethnology" OR "ethnol*" OR "Indians" OR "Asians" OR "Asian Americans" OR "Blacks" OR "African Americans" OR "Whites" OR "Hispanics" OR "Latino" OR "Mexican Americans" OR "Indigenous" OR "ancestry" OR "migrant" OR "undocumented immigrant" OR "Emigrants" OR "Emigrant" OR "emigrat*" OR "Immigrants" OR "Immigrant" OR "immigrat*" OR "Refugee" OR "Refugees" OR "Refugee" OR "Asylum Seekers" OR "Asylum Seeker" OR "Displaced Persons" OR "Displaced Person" OR "sociodemographic factor" OR "sociodemographic factors" OR "socio demographic factor" OR "socio demographic factors") OR AB=("Ethnicity" OR "Ethnology" OR "ethnic or racial aspects" OR "Ethnic Group" OR "Population Group" OR "Population Groups" OR "Population Group" OR "ethnic" OR "ethnicity" OR "ethnic*" OR "ethnology" OR "ethnol*" OR "Indians" OR "Asians" OR "Asian Americans" OR "Blacks" OR "African Americans" OR "Whites" OR "Hispanics" OR "Latino" OR "Mexican Americans" OR "Indigenous" OR "ancestry" OR "migrant" OR "undocumented immigrant" OR "Emigrants" OR "Emigrant" OR "emigrat*" OR "Immigrants" OR "Immigrant" OR "immigrat*" OR "Refugee" OR "Refugees" OR "Refugee" OR "Asylum Seekers" OR "Asylum Seeker" OR "Displaced Persons" OR "Displaced Person" OR "sociodemographic factor" OR "sociodemographic factors" OR "socio demographic factor" OR "socio demographic factors")) AND (TI=("Quality of Life" OR "Quality of Life" OR "QoL" OR "HRQoL" OR "life quality" OR "Sickness Impact Profile" OR "Sickness Impact Profile") OR AK=("Quality of Life" OR "Quality of Life" OR "QoL" OR "HRQoL" OR "life quality" OR "Sickness Impact Profile" OR "Sickness Impact Profile") OR AB=("Quality of Life" OR "Quality of Life" OR "QoL" OR "HRQoL" OR "life quality" OR "Sickness Impact Profile" OR

"Sickness Impact Profile"))) **OR** ((TI=("cerebrovascular accident" OR "Stroke" OR "strokes" OR "CVA" OR "CVAs" OR "Cerebrovascular Accident" OR "Cerebrovascular Accidents" OR "Brain Attack" OR "Brain Attacks" OR "Brain Infarction" OR "Brain Infarctions" OR "Brain Infarct*" OR "Brain Infarction" OR "Cerebrovascular Apoplexy" OR "Apoplexy" OR "Brain Vascular Accident" OR "Brain Vascular Accidents" OR "Cerebrovascular Stroke" OR "Cerebrovascular Strokes" OR "Cerebral Stroke" OR "Cerebral Strokes" OR "Acute Stroke" OR "Acute Strokes" OR "Acute Cerebrovascular Accident" OR "Acute Cerebrovascular Accidents" OR "Cerebrovascular Event" OR "Cerebrovascular Events" OR "Cerebrovascular Attack" OR "Cerebrovascular Attacks" OR "poststroke" OR "Cerebral infarction" OR "Cerebral infarctions" OR "Cerebral infarct*" OR "Brain ischemia" OR "Brain ischemia" OR "Brain ischaemia" OR "Cerebral Ischemia" OR "Cerebral Ischaemia" OR "Brain Hemorrhage" OR Cerebral Hemorrhag* OR Cerebral Haemorrhag* OR Intracerebral Hemorrhag* OR Brain Hemorrhag* OR Brain Haemorrhag* OR Intracerebral Haemorrhag* OR "Neurologic Event" OR "Neurologic Events" OR "Transient Ischemic Attack" OR "transient ischemic attack" OR "transient ischemic attack" OR "transient ischaemic attack" OR "transient ischaemic attacks" OR "TIA" OR "TIAs" OR "transient brainstem ischemia" OR "transient brainstem ischaemia" OR "transient brain stem ischemia" OR "transient brain ischemia" OR "transient brain ischaemia" OR "transient cerebral ischemia" OR "transient cerebral ischaemia" OR "transient cerebral ischemic" OR "transient cerebral ischaemic" OR ("transient" AND ("brain" OR "cerebral") AND (ischemi* OR ischaem*))) OR AK=("cerebrovascular accident" OR "Stroke" OR "strokes" OR "CVA" OR "CVAs" OR "Cerebrovascular Accident" OR "Cerebrovascular Accidents" OR "Brain Attack" OR "Brain Attacks" OR "Brain Infarction" OR "Brain Infarctions" OR "Brain Infarct*" OR "Brain Infarction" OR "Cerebrovascular Apoplexy" OR "Apoplexy" OR "Brain Vascular Accident" OR "Brain Vascular Accidents" OR "Cerebrovascular Stroke" OR "Cerebrovascular Strokes" OR "Cerebral Stroke" OR "Cerebral Strokes" OR "Acute Stroke" OR "Acute Strokes" OR "Acute Cerebrovascular Accident" OR "Acute Cerebrovascular Accidents" OR "Cerebrovascular Event" OR "Cerebrovascular Events" OR "Cerebrovascular Attack" OR "Cerebrovascular Attacks" OR "poststroke" OR "Cerebral infarction" OR "Cerebral infarctions" OR "Cerebral infarct*" OR "Brain ischemia" OR "Brain ischemia" OR "Brain ischaemia" OR "Cerebral Ischemia" OR "Cerebral Ischaemia" OR "Brain Hemorrhage" OR Cerebral Hemorrhag* OR Cerebral Haemorrhag* OR Intracerebral Hemorrhag* OR Brain Hemorrhag* OR Brain Haemorrhag* OR Intracerebral Haemorrhag* OR "Neurologic Event" OR "Neurologic Events" OR "Transient Ischemic Attack" OR "transient ischemic attack" OR "transient ischemic attack" OR "transient ischaemic attack" OR "transient ischaemic attacks" OR "TIA" OR "TIAs" OR "transient brainstem ischemia" OR "transient brainstem ischaemia" OR "transient brain stem ischemia" OR "transient brain ischemia" OR "transient brain ischaemia" OR "transient cerebral ischemia" OR "transient cerebral ischaemia" OR "transient cerebral ischemic" OR "transient cerebral ischaemic" OR ("transient" AND ("brain" OR "cerebral") AND (ischemi* OR ischaem*))) OR AB=("cerebrovascular accident" OR "Stroke" OR "strokes" OR "CVA" OR "CVAs" OR "Cerebrovascular Accident" OR "Cerebrovascular Accidents" OR "Brain Attack" OR "Brain Attacks" OR "Brain Infarction" OR "Brain Infarctions" OR "Brain Infarct*" OR "Brain Infarction" OR "Cerebrovascular Apoplexy" OR "Apoplexy" OR "Brain Vascular Accident" OR "Brain Vascular Accidents" OR "Cerebrovascular Stroke" OR "Cerebrovascular Strokes" OR "Cerebral Stroke" OR "Cerebral Strokes" OR "Acute Stroke" OR "Acute Strokes" OR "Acute Cerebrovascular Accident" OR "Acute Cerebrovascular Accidents" OR "Cerebrovascular Event" OR "Cerebrovascular Events" OR "Cerebrovascular Attack" OR "Cerebrovascular Attacks" OR "poststroke" OR "Cerebral infarction" OR "Cerebral infarctions" OR "Cerebral infarct*" OR "Brain ischemia" OR "Brain ischemia" OR "Brain ischaemia" OR "Cerebral Ischemia" OR "Cerebral Ischaemia" OR "Brain Hemorrhage" OR Cerebral Hemorrhag* OR Cerebral Haemorrhag* OR Intracerebral Hemorrhag* OR Brain Hemorrhag* OR Brain Haemorrhag* OR Intracerebral Haemorrhag* OR "Neurologic Event" OR "Neurologic Events" OR "Transient Ischemic Attack" OR "transient ischemic attack" OR "transient ischemic attack" OR "transient ischaemic attack" OR "transient ischaemic attacks" OR "TIA" OR "TIAs" OR "transient brainstem ischemia"

OR "transient brainstem ischaemia" OR "transient brain stem ischemia" OR "transient brain ischemia" OR "transient brain ischaemia" OR "transient cerebral ischemia" OR "transient cerebral ischaemia" OR "transient cerebral ischemic" OR "transient cerebral ischaemic" OR ("transient" AND ("brain" OR "cerebral") AND (ischemi* OR ischaem*)))) AND TI=("Ethnicity" OR "Ethnology" OR "ethnic or racial aspects" OR "Ethnic Group" OR "Population Group" OR "Population Groups" OR "Population Group" OR "ethnic" OR "ethnicity" OR "ethnic*" OR "ethnology" OR "ethnol*" OR "Indians" OR "Asians" OR "Asian Americans" OR "Blacks" OR "African Americans" OR "Whites" OR "Hispanics" OR "Latino" OR "Mexican Americans" OR "Indigenous" OR "ancestry" OR "migrant" OR "undocumented immigrant" OR "Emigrants" OR "Emigrant" OR "emigrat*" OR "Immigrants" OR "Immigrant" OR "immigrat*" OR "Refugee" OR "Refugees" OR "Refugee" OR "Asylum Seekers" OR "Asylum Seeker" OR "Displaced Persons" OR "Displaced Person" OR "sociodemographic factor" OR "sociodemographic factors" OR "socio demographic factor" OR "socio demographic factors") AND (TI=("Quality of Life" OR "Quality of Life" OR "QoL" OR "HRQoL" OR "life quality" OR "Sickness Impact Profile" OR "Sickness Impact Profile") OR AK=("Quality of Life" OR "Quality of Life" OR "QoL" OR "HRQoL" OR "life quality" OR "Sickness Impact Profile" OR "Sickness Impact Profile") OR AB=("Quality of Life" OR "Quality of Life" OR "QoL" OR "HRQoL" OR "life quality" OR "Sickness Impact Profile" OR "Sickness Impact Profile")))) NOT DT=(meeting abstract)

Cochrane

(("cerebrovascular accident" OR "Stroke" OR "strokes" OR "CVA" OR "CVAs" OR "Cerebrovascular Accident" OR "Cerebrovascular Accidents" OR "Brain Attack" OR "Brain Attacks" OR "Brain Infarction" OR "Brain Infarctions" OR "Brain Infarct*" OR "Brain Infarction" OR "Cerebrovascular Apoplexy" OR "Apoplexy" OR "Brain Vascular Accident" OR "Brain Vascular Accidents" OR "Cerebrovascular Stroke" OR "Cerebrovascular Strokes" OR "Cerebral Stroke" OR "Cerebral Strokes" OR "Acute Stroke" OR "Acute Strokes" OR "Acute Cerebrovascular Accident" OR "Acute Cerebrovascular Accidents" OR "Cerebrovascular Event" OR "Cerebrovascular Events" OR "Cerebrovascular Attack" OR "Cerebrovascular Attacks" OR "poststroke" OR "Cerebral infarction" OR "Cerebral infarctions" OR "Cerebral infarct*" OR "Brain ischemia" OR "Brain ischemia" OR "Brain ischaemia" OR "Cerebral Ischemia" OR "Cerebral Ischaemia" OR "Brain Hemorrhage" OR Cerebral Hemorrhag* OR Cerebral Haemorrhag* OR Intracerebral Hemorrhag* OR Brain Hemorrhag* OR Brain Haemorrhag* OR Intracerebral Haemorrhag* OR "Neurologic Event" OR "Neurologic Events" OR "Transient Ischemic Attack" OR "transient ischemic attack" OR "transient ischemic attack" OR "transient ischaemic attack" OR "transient ischaemic attacks" OR "TIA" OR "TIAs" OR "transient brainstem ischemia" OR "transient brainstem ischaemia" OR "transient brain stem ischemia" OR "transient brain ischemia" OR "transient brain ischaemia" OR "transient cerebral ischemia" OR "transient cerebral ischaemia" OR "transient cerebral ischemic" OR "transient cerebral ischaemic" OR ("transient" AND ("brain" OR "cerebral") AND (ischemi* OR ischaem*))) AND ("Ethnicity" OR "Ethnology" OR "ethnic or racial aspects" OR "Ethnic Group" OR "Population Group" OR "Population Groups" OR "Population Group" OR "ethnic" OR "ethnicity" OR "ethnic*" OR "ethnology" OR "ethnol*" OR "Indians" OR "Asians" OR "Asian Americans" OR "Blacks" OR "African Americans" OR "Whites" OR "Hispanics" OR "Latino" OR "Mexican Americans" OR "Indigenous" OR "ancestry" OR "migrant" OR "undocumented immigrant" OR "Emigrants" OR "Emigrant" OR "emigrat*" OR "Immigrants" OR "Immigrant" OR "immigrat*" OR "Refugee" OR "Refugees" OR "Refugee" OR "Asylum Seekers" OR "Asylum Seeker" OR "Displaced Persons" OR "Displaced Person" OR "sociodemographic factor" OR "sociodemographic factors" OR "socio demographic factor" OR "socio demographic factors") AND ("Quality of Life" OR "Quality of Life" OR "QoL" OR "HRQoL" OR "life quality" OR "Sickness Impact Profile" OR "Sickness Impact Profile")):ti,kw **OR** (("cerebrovascular accident" OR "Stroke" OR "strokes" OR "CVA" OR "CVAs" OR "Cerebrovascular Accident" OR "Cerebrovascular Accidents" OR "Brain Attack" OR "Brain Attacks" OR "Brain Infarction" OR "Brain Infarctions" OR "Brain Infarct*" OR "Brain Infarction" OR "Cerebrovascular Apoplexy" OR "Apoplexy" OR "Brain Vascular Accident" OR "Brain Vascular Accidents" OR "Cerebrovascular Stroke" OR "Cerebrovascular Strokes" OR "Cerebral Stroke" OR "Cerebral Strokes" OR "Acute Stroke" OR "Acute Strokes" OR "Acute Cerebrovascular Accident" OR "Acute Cerebrovascular Accidents" OR "Cerebrovascular Event" OR "Cerebrovascular Events" OR "Cerebrovascular Attack" OR "Cerebrovascular Attacks" OR "poststroke" OR "Cerebral infarction" OR "Cerebral infarctions" OR "Cerebral infarct*" OR "Brain ischemia" OR "Brain ischemia" OR "Brain ischaemia" OR "Cerebral Ischemia" OR "Cerebral Ischaemia" OR "Brain Hemorrhage" OR Cerebral Hemorrhag* OR Cerebral Haemorrhag* OR Intracerebral Hemorrhag* OR Brain Hemorrhag* OR Brain Haemorrhag* OR Intracerebral Haemorrhag* OR "Neurologic Event" OR "Neurologic Events" OR "Transient Ischemic Attack" OR "transient ischemic attack" OR "transient ischemic attack" OR "transient ischaemic attack" OR "transient ischaemic attacks" OR "TIA" OR "TIAs" OR "transient brainstem ischemia" OR "transient brainstem ischaemia" OR "transient brain stem ischemia" OR "transient brain ischemia" OR "transient brain ischaemia" OR "transient cerebral ischemia" OR "transient cerebral ischaemia" OR "transient cerebral ischemic" OR "transient cerebral ischaemic" OR ("transient" AND ("brain" OR "cerebral") AND (ischemi* OR ischaem*))):ti AND ("Ethnicity" OR "Ethnology" OR "ethnic or racial aspects" OR "Ethnic Group" OR "Population Group" OR "Population Groups" OR "Population Group" OR "ethnic" OR "ethnicity" OR "ethnic*" OR "ethnology" OR "ethnol*" OR "Indians" OR

"Asians" OR "Asian Americans" OR "Blacks" OR "African Americans" OR "Whites" OR "Hispanics" OR "Latino" OR "Mexican Americans" OR "Indigenous" OR "ancestry" OR "migrant" OR "undocumented immigrant" OR "Emigrants" OR "Emigrant" OR "emigrat*" OR "Immigrants" OR "Immigrant" OR "immigrat*" OR "Refugee" OR "Refugees" OR "Refugee" OR "Asylum Seekers" OR "Asylum Seeker" OR "Displaced Persons" OR "Displaced Person" OR "sociodemographic factor" OR "sociodemographic factors" OR "socio demographic factor" OR "socio demographic factors"):ti,ab,kw AND ("Quality of Life" OR "Quality of Life" OR "QoL" OR "HRQoL" OR "life quality" OR "Sickness Impact Profile" OR "Sickness Impact Profile"):ti,ab,kw)

Emcare

(((exp *"cerebrovascular accident"/ OR "Stroke".ti OR "strokes".ti OR "CVA".ti OR "CVAs".ti OR "Cerebrovascular Accident".ti OR "Cerebrovascular Accidents".ti OR "Brain Attack".ti OR "Brain Attacks".ti OR "Brain Infarction".ti OR "Brain Infarctions".ti OR "Brain Infarct*".ti OR exp *"Brain Infarction"/ OR "Cerebrovascular Apoplexy".ti OR "Apoplexy".ti OR "Brain Vascular Accident".ti OR "Brain Vascular Accidents".ti OR "Cerebrovascular Stroke".ti OR "Cerebrovascular Strokes".ti OR "Cerebral Stroke".ti OR "Cerebral Strokes".ti OR "Acute Stroke".ti OR "Acute Strokes".ti OR "Acute Cerebrovascular Accident".ti OR "Acute Cerebrovascular Accidents".ti OR "Cerebrovascular Event".ti OR "Cerebrovascular Events".ti OR "Cerebrovascular Attack".ti OR "Cerebrovascular Attacks".ti OR "poststroke".ti OR "Cerebral infarction".ti OR "Cerebral infarctions".ti OR "Cerebral infarct*".ti OR exp *"Brain ischemia"/ OR "Brain ischemia".ti OR "Brain ischaemia".ti OR "Cerebral Ischemia".ti OR "Cerebral Ischaemia".ti OR exp *"Brain Hemorrhage"/ OR Cerebral Hemorrhag*.ti OR Cerebral Haemorrhag*.ti OR Intracerebral Hemorrhag*.ti OR Brain Hemorrhag*.ti OR Brain Haemorrhag*.ti OR Intracerebral Haemorrhag*.ti OR "Neurologic Event".ti OR "Neurologic Events".ti OR exp *"Transient Ischemic Attack"/ OR "transient ischemic attack".ti OR "transient ischemic attack".ti OR "transient ischaemic attack".ti OR "transient ischaemic attacks".ti OR "TIA".ti OR "TIAs".ti OR "transient brainstem ischemia".ti OR "transient brainstem ischaemia".ti OR "transient brain stem ischemia".ti OR "transient brain ischemia".ti OR "transient brain ischaemia".ti OR "transient cerebral ischemia".ti OR "transient cerebral ischaemia".ti OR "transient cerebral ischemic".ti OR "transient cerebral ischaemic".ti OR ("transient".ti AND ("brain".ti OR "cerebral".ti) AND (ischemi*.ti OR ischaem*.ti))) AND (exp *"Ethnicity"/ OR exp *"Ethnology"/ OR exp *"ethnic or racial aspects"/ OR exp *"Ethnic Group"/ OR exp *"Population Group"/ OR "Population Groups".ti,ab OR "Population Group".ti,ab OR "ethnic".ti,ab OR "ethnicity".ti,ab OR "ethnic*".ti,ab OR "ethnology".ti,ab OR "ethnol*".ti,ab OR "Indians".ti,ab OR "Asians".ti,ab OR "Asian Americans".ti,ab OR "Blacks".ti,ab OR "African Americans".ti,ab OR "Whites".ti,ab OR "Hispanics".ti,ab OR "Latino".ti,ab OR "Mexican Americans".ti,ab OR "Indigenous".ti,ab OR "ancestry".ti,ab OR exp *"migrant"/ OR exp *"undocumented immigrant"/ OR "Emigrants".ti,ab OR "Emigrant".ti,ab OR "emigrat*".ti,ab OR "Immigrants".ti,ab OR "Immigrant".ti,ab OR "immigrat*".ti,ab OR exp *"Refugee"/ OR "Refugees".ti,ab OR "Refugee".ti,ab OR "Asylum Seekers".ti,ab OR "Asylum Seeker".ti,ab OR "Displaced Persons".ti,ab OR "Displaced Person".ti,ab OR "sociodemographic factor".ti,ab OR "sociodemographic factors".ti,ab OR "socio demographic factor".ti,ab OR "socio demographic factors".ti,ab) AND (exp *"Quality of Life"/ OR "Quality of Life".ti,ab OR "QoL".ti,ab OR "HRQoL".ti,ab OR "life quality".ti,ab OR exp *"Sickness Impact Profile"/ OR "Sickness Impact Profile".ti,ab)) **OR** ((exp *"cerebrovascular accident"/ OR "Stroke".ti,ab OR "strokes".ti,ab OR "CVA".ti,ab OR "CVAs".ti,ab OR "Cerebrovascular Accident".ti,ab OR "Cerebrovascular Accidents".ti,ab OR "Brain Attack".ti,ab OR "Brain Attacks".ti,ab OR "Brain Infarction".ti,ab OR "Brain Infarctions".ti,ab OR "Brain Infarct*".ti,ab OR exp *"Brain Infarction"/ OR "Cerebrovascular Apoplexy".ti,ab OR "Apoplexy".ti,ab OR "Brain Vascular Accident".ti,ab OR "Brain Vascular Accidents".ti,ab OR "Cerebrovascular Stroke".ti,ab OR "Cerebrovascular Strokes".ti,ab OR "Cerebral Stroke".ti,ab OR "Cerebral Strokes".ti,ab OR "Acute Stroke".ti,ab OR "Acute Strokes".ti,ab OR "Acute Cerebrovascular Accident".ti,ab OR "Acute Cerebrovascular Accidents".ti,ab OR "Cerebrovascular Event".ti,ab OR "Cerebrovascular Events".ti,ab OR "Cerebrovascular Attack".ti,ab OR "Cerebrovascular Attacks".ti,ab OR "poststroke".ti,ab OR "Cerebral infarction".ti,ab OR "Cerebral infarctions".ti,ab OR "Cerebral infarct*".ti,ab OR exp *"Brain ischemia"/ OR "Brain ischemia".ti,ab OR "Brain ischaemia".ti,ab OR "Cerebral Ischemia".ti,ab OR "Cerebral Ischaemia".ti,ab OR exp *"Brain Hemorrhage"/ OR Cerebral Hemorrhag*.ti,ab OR Cerebral Haemorrhag*.ti,ab OR Intracerebral Hemorrhag*.ti,ab OR Brain Hemorrhag*.ti,ab OR Brain Haemorrhag*.ti,ab OR Intracerebral Haemorrhag*.ti,ab OR "Neurologic Event".ti,ab OR "Neurologic Events".ti,ab OR exp *"Transient Ischemic Attack"/ OR "transient ischemic attack".ti,ab OR "transient ischemic attack".ti,ab OR "transient ischaemic attack".ti,ab OR "transient

ischaemic attacks".ti,ab OR "TIA".ti,ab OR "TIAs".ti,ab OR "transient brainstem ischemia".ti,ab OR "transient brainstem ischaemia".ti,ab OR "transient brain stem ischemia".ti,ab OR "transient brain ischemia".ti,ab OR "transient brain ischaemia".ti,ab OR "transient cerebral ischemia".ti,ab OR "transient cerebral ischaemia".ti,ab OR "transient cerebral ischemic".ti,ab OR "transient cerebral ischaemic".ti,ab OR ("transient".ti,ab AND ("brain".ti,ab OR "cerebral".ti,ab) AND (ischemi*.ti,ab OR ischaem*.ti,ab))) AND (exp *"Ethnicity"/ OR exp *"Ethnology"/ OR exp *"ethnic or racial aspects"/ OR exp *"Ethnic Group"/ OR exp *"Population Group"/ OR "Population Groups".ti OR "Population Group".ti OR "ethnic".ti OR "ethnicity".ti OR "ethnic*".ti OR "ethnology".ti OR "ethnol*".ti OR "Indians".ti OR "Asians".ti OR "Asian Americans".ti OR "Blacks".ti OR "African Americans".ti OR "Whites".ti OR "Hispanics".ti OR "Latino".ti OR "Mexican Americans".ti OR "Indigenous".ti OR "ancestry".ti OR exp *"migrant"/ OR exp *"undocumented immigrant"/ OR "Emigrants".ti OR "Emigrant".ti OR "emigrat*".ti OR "Immigrants".ti OR "Immigrant".ti OR "immigrat*".ti OR exp *"Refugee"/ OR "Refugees".ti OR "Refugee".ti OR "Asylum Seekers".ti OR "Asylum Seeker".ti OR "Displaced Persons".ti OR "Displaced Person".ti OR "sociodemographic factor".ti OR "sociodemographic factors".ti OR "socio demographic factor".ti OR "socio demographic factors".ti) AND (exp *"Quality of Life"/ OR "Quality of Life".ti,ab OR "QoL".ti,ab OR "HRQoL".ti,ab OR "life quality".ti,ab OR exp *"Sickness Impact Profile"/ OR "Sickness Impact Profile".ti,ab)))

PsycINFO

((TI("cerebrovascular accident" OR "Stroke" OR "strokes" OR "CVA" OR "CVAs" OR "Cerebrovascular Accident" OR "Cerebrovascular Accidents" OR "Brain Attack" OR "Brain Attacks" OR "Brain Infarction" OR "Brain Infarctions" OR "Brain Infarct*" OR "Brain Infarction" OR "Cerebrovascular Apoplexy" OR "Apoplexy" OR "Brain Vascular Accident" OR "Brain Vascular Accidents" OR "Cerebrovascular Stroke" OR "Cerebrovascular Strokes" OR "Cerebral Stroke" OR "Cerebral Strokes" OR "Acute Stroke" OR "Acute Strokes" OR "Acute Cerebrovascular Accident" OR "Acute Cerebrovascular Accidents" OR "Cerebrovascular Event" OR "Cerebrovascular Events" OR "Cerebrovascular Attack" OR "Cerebrovascular Attacks" OR "poststroke" OR "Cerebral infarction" OR "Cerebral infarctions" OR "Cerebral infarct*" OR "Brain ischemia" OR "Brain ischemia" OR "Brain ischaemia" OR "Cerebral Ischemia" OR "Cerebral Ischaemia" OR "Brain Hemorrhage" OR Cerebral Hemorrhag* OR Cerebral Haemorrhag* OR Intracerebral Hemorrhag* OR Brain Hemorrhag* OR Brain Haemorrhag* OR Intracerebral Haemorrhag* OR "Neurologic Event" OR "Neurologic Events" OR "Transient Ischemic Attack" OR "transient ischemic attack" OR "transient ischemic attack" OR "transient ischaemic attack" OR "transient ischaemic attacks" OR "TIA" OR "TIAs" OR "transient brainstem ischemia" OR "transient brainstem ischaemia" OR "transient brain stem ischemia" OR "transient brain ischemia" OR "transient brain ischaemia" OR "transient cerebral ischemia" OR "transient cerebral ischaemia" OR "transient cerebral ischemic" OR "transient cerebral ischaemic" OR ("transient" AND ("brain" OR "cerebral") AND (ischemi* OR ischaem*))) AND (TI("Ethnicity" OR "Ethnology" OR "ethnic or racial aspects" OR "Ethnic Group" OR "Population Group" OR "Population Groups" OR "Population Group" OR "ethnic" OR "ethnicity" OR "ethnic*" OR "ethnology" OR "ethnol*" OR "Indians" OR "Asians" OR "Asian Americans" OR "Blacks" OR "African Americans" OR "Whites" OR "Hispanics" OR "Latino" OR "Mexican Americans" OR "Indigenous" OR "ancestry" OR "migrant" OR "undocumented immigrant" OR "Emigrants" OR "Emigrant" OR "emigrat*" OR "Immigrants" OR "Immigrant" OR "immigrat*" OR "Refugee" OR "Refugees" OR "Refugee" OR "Asylum Seekers" OR "Asylum Seeker" OR "Displaced Persons" OR "Displaced Person" OR "sociodemographic factor" OR "sociodemographic factors" OR "socio demographic factor" OR "socio demographic factors") OR SU("Ethnicity" OR "Ethnology" OR "ethnic or racial aspects" OR "Ethnic Group" OR "Population Group" OR "Population Groups" OR "Population Group" OR "ethnic" OR "ethnicity" OR "ethnic*" OR "ethnology" OR "ethnol*" OR "Indians" OR "Asians" OR "Asian Americans" OR "Blacks" OR "African Americans" OR "Whites" OR "Hispanics" OR "Latino" OR "Mexican Americans" OR "Indigenous" OR "ancestry" OR "migrant" OR "undocumented immigrant" OR "Emigrants" OR "Emigrant" OR "emigrat*" OR "Immigrants" OR "Immigrant" OR "immigrat*" OR "Refugee" OR "Refugees" OR "Refugee" OR "Asylum Seekers" OR "Asylum Seeker" OR "Displaced Persons" OR "Displaced Person" OR "sociodemographic factor" OR "sociodemographic factors" OR "socio demographic factor" OR "socio demographic factors") OR AB("Ethnicity" OR "Ethnology" OR "ethnic or racial aspects" OR "Ethnic Group" OR "Population Group" OR "Population Groups" OR "Population Group" OR "ethnic" OR "ethnicity" OR "ethnic*" OR "ethnology" OR "ethnol*" OR "Indians" OR "Asians" OR "Asian Americans" OR "Blacks" OR "African Americans" OR "Whites" OR "Hispanics" OR "Latino" OR "Mexican Americans" OR "Indigenous" OR "ancestry" OR "migrant" OR "undocumented immigrant" OR "Emigrants" OR "Emigrant" OR "emigrat*" OR "Immigrants" OR "Immigrant" OR "immigrat*" OR "Refugee" OR "Refugees" OR "Refugee" OR "Asylum Seekers" OR "Asylum Seeker" OR "Displaced Persons" OR "Displaced Person" OR "sociodemographic factor" OR "sociodemographic factors" OR "socio demographic factor" OR "socio demographic factors") OR MA("Ethnicity" OR "Ethnology" OR "ethnic or racial aspects" OR "Ethnic Group" OR "Population Group" OR "Population Groups" OR "Population Group" OR "ethnic" OR "ethnicity" OR "ethnic*" OR "ethnology" OR "ethnol*" OR "Indians" OR "Asians" OR "Asian Americans" OR "Blacks" OR "African Americans" OR "Whites" OR "Hispanics" OR "Latino" OR "Mexican Americans" OR "Indigenous" OR "ancestry" OR

"migrant" OR "undocumented immigrant" OR "Emigrants" OR "Emigrant" OR "emigrat*" OR "Immigrants" OR "Immigrant" OR "immigrat*" OR "Refugee" OR "Refugees" OR "Refugee" OR "Asylum Seekers" OR "Asylum Seeker" OR "Displaced Persons" OR "Displaced Person" OR "sociodemographic factor" OR "sociodemographic factors" OR "socio demographic factor" OR "socio demographic factors")) AND (TI("Quality of Life" OR "Quality of Life" OR "QoL" OR "HRQoL" OR "life quality" OR "Sickness Impact Profile" OR "Sickness Impact Profile") OR SU("Quality of Life" OR "Quality of Life" OR "QoL" OR "HRQoL" OR "life quality" OR "Sickness Impact Profile" OR "Sickness Impact Profile") OR AB("Quality of Life" OR "Quality of Life" OR "QoL" OR "HRQoL" OR "life quality" OR "Sickness Impact Profile" OR "Sickness Impact Profile") OR MA("Quality of Life" OR "Quality of Life" OR "QoL" OR "HRQoL" OR "life quality" OR "Sickness Impact Profile" OR "Sickness Impact Profile"))) **OR** ((TI("cerebrovascular accident" OR "Stroke" OR "strokes" OR "CVA" OR "CVAs" OR "Cerebrovascular Accident" OR "Cerebrovascular Accidents" OR "Brain Attack" OR "Brain Attacks" OR "Brain Infarction" OR "Brain Infarctions" OR "Brain Infarct*" OR "Brain Infarction" OR "Cerebrovascular Apoplexy" OR "Apoplexy" OR "Brain Vascular Accident" OR "Brain Vascular Accidents" OR "Cerebrovascular Stroke" OR "Cerebrovascular Strokes" OR "Cerebral Stroke" OR "Cerebral Strokes" OR "Acute Stroke" OR "Acute Strokes" OR "Acute Cerebrovascular Accident" OR "Acute Cerebrovascular Accidents" OR "Cerebrovascular Event" OR "Cerebrovascular Events" OR "Cerebrovascular Attack" OR "Cerebrovascular Attacks" OR "poststroke" OR "Cerebral infarction" OR "Cerebral infarctions" OR "Cerebral infarct*" OR "Brain ischemia" OR "Brain ischemia" OR "Brain ischaemia" OR "Cerebral Ischemia" OR "Cerebral Ischaemia" OR "Brain Hemorrhage" OR Cerebral Hemorrhag* OR Cerebral Haemorrhag* OR Intracerebral Hemorrhag* OR Brain Hemorrhag* OR Brain Haemorrhag* OR Intracerebral Haemorrhag* OR "Neurologic Event" OR "Neurologic Events" OR "Transient Ischemic Attack" OR "transient ischemic attack" OR "transient ischemic attack" OR "transient ischaemic attack" OR "transient ischaemic attacks" OR "TIA" OR "TIAs" OR "transient brainstem ischemia" OR "transient brainstem ischaemia" OR "transient brain stem ischemia" OR "transient brain ischemia" OR "transient brain ischaemia" OR "transient cerebral ischemia" OR "transient cerebral ischaemia" OR "transient cerebral ischemic" OR "transient cerebral ischaemic" OR ("transient" AND ("brain" OR "cerebral") AND (ischemi* OR ischaem*))) OR SU("cerebrovascular accident" OR "Stroke" OR "strokes" OR "CVA" OR "CVAs" OR "Cerebrovascular Accident" OR "Cerebrovascular Accidents" OR "Brain Attack" OR "Brain Attacks" OR "Brain Infarction" OR "Brain Infarctions" OR "Brain Infarct*" OR "Brain Infarction" OR "Cerebrovascular Apoplexy" OR "Apoplexy" OR "Brain Vascular Accident" OR "Brain Vascular Accidents" OR "Cerebrovascular Stroke" OR "Cerebrovascular Strokes" OR "Cerebral Stroke" OR "Cerebral Strokes" OR "Acute Stroke" OR "Acute Strokes" OR "Acute Cerebrovascular Accident" OR "Acute Cerebrovascular Accidents" OR "Cerebrovascular Event" OR "Cerebrovascular Events" OR "Cerebrovascular Attack" OR "Cerebrovascular Attacks" OR "poststroke" OR "Cerebral infarction" OR "Cerebral infarctions" OR "Cerebral infarct*" OR "Brain ischemia" OR "Brain ischemia" OR "Brain ischaemia" OR "Cerebral Ischemia" OR "Cerebral Ischaemia" OR "Brain Hemorrhage" OR Cerebral Hemorrhag* OR Cerebral Haemorrhag* OR Intracerebral Hemorrhag* OR Brain Hemorrhag* OR Brain Haemorrhag* OR Intracerebral Haemorrhag* OR "Neurologic Event" OR "Neurologic Events" OR "Transient Ischemic Attack" OR "transient ischemic attack" OR "transient ischemic attack" OR "transient ischaemic attack" OR "transient ischaemic attacks" OR "TIA" OR "TIAs" OR "transient brainstem ischemia" OR "transient brainstem ischaemia" OR "transient brain stem ischemia" OR "transient brain ischemia" OR "transient brain ischaemia" OR "transient cerebral ischemia" OR "transient cerebral ischaemia" OR "transient cerebral ischemic" OR "transient cerebral ischaemic" OR ("transient" AND ("brain" OR "cerebral") AND (ischemi* OR ischaem*))) OR AB("cerebrovascular accident" OR "Stroke" OR "strokes" OR "CVA" OR "CVAs" OR "Cerebrovascular Accident" OR "Cerebrovascular Accidents" OR "Brain Attack" OR "Brain Attacks" OR "Brain Infarction" OR "Brain Infarctions" OR "Brain Infarct*" OR "Brain Infarction" OR "Cerebrovascular Apoplexy" OR "Apoplexy" OR "Brain Vascular Accident" OR "Brain Vascular Accidents" OR "Cerebrovascular Stroke" OR

"Cerebrovascular Strokes" OR "Cerebral Stroke" OR "Cerebral Strokes" OR "Acute Stroke" OR "Acute Strokes" OR "Acute Cerebrovascular Accident" OR "Acute Cerebrovascular Accidents" OR "Cerebrovascular Event" OR "Cerebrovascular Events" OR "Cerebrovascular Attack" OR "Cerebrovascular Attacks" OR "poststroke" OR "Cerebral infarction" OR "Cerebral infarctions" OR "Cerebral infarct*" OR "Brain ischemia" OR "Brain ischemia" OR "Brain ischaemia" OR "Cerebral Ischemia" OR "Cerebral Ischaemia" OR "Brain Hemorrhage" OR Cerebral Hemorrhag* OR Cerebral Haemorrhag* OR Intracerebral Hemorrhag* OR Brain Hemorrhag* OR Brain Haemorrhag* OR Intracerebral Haemorrhag* OR "Neurologic Event" OR "Neurologic Events" OR "Transient Ischemic Attack" OR "transient ischemic attack" OR "transient ischemic attack" OR "transient ischaemic attack" OR "transient ischaemic attacks" OR "TIA" OR "TIAs" OR "transient brainstem ischemia" OR "transient brainstem ischaemia" OR "transient brain stem ischemia" OR "transient brain ischemia" OR "transient brain ischaemia" OR "transient cerebral ischemia" OR "transient cerebral ischaemia" OR "transient cerebral ischemic" OR "transient cerebral ischaemic" OR ("transient" AND ("brain" OR "cerebral") AND (ischemi* OR ischaem*))) OR MA("cerebrovascular accident" OR "Stroke" OR "strokes" OR "CVA" OR "CVAs" OR "Cerebrovascular Accident" OR "Cerebrovascular Accidents" OR "Brain Attack" OR "Brain Attacks" OR "Brain Infarction" OR "Brain Infarctions" OR "Brain Infarct*" OR "Brain Infarction" OR "Cerebrovascular Apoplexy" OR "Apoplexy" OR "Brain Vascular Accident" OR "Brain Vascular Accidents" OR "Cerebrovascular Stroke" OR "Cerebrovascular Strokes" OR "Cerebral Stroke" OR "Cerebral Strokes" OR "Acute Stroke" OR "Acute Strokes" OR "Acute Cerebrovascular Accident" OR "Acute Cerebrovascular Accidents" OR "Cerebrovascular Event" OR "Cerebrovascular Events" OR "Cerebrovascular Attack" OR "Cerebrovascular Attacks" OR "poststroke" OR "Cerebral infarction" OR "Cerebral infarctions" OR "Cerebral infarct*" OR "Brain ischemia" OR "Brain ischemia" OR "Brain ischaemia" OR "Cerebral Ischemia" OR "Cerebral Ischaemia" OR "Brain Hemorrhage" OR Cerebral Hemorrhag* OR Cerebral Haemorrhag* OR Intracerebral Hemorrhag* OR Brain Hemorrhag* OR Brain Haemorrhag* OR Intracerebral Haemorrhag* OR "Neurologic Event" OR "Neurologic Events" OR "Transient Ischemic Attack" OR "transient ischemic attack" OR "transient ischemic attack" OR "transient ischaemic attack" OR "transient ischaemic attacks" OR "TIA" OR "TIAs" OR "transient brainstem ischemia" OR "transient brainstem ischaemia" OR "transient brain stem ischemia" OR "transient brain ischemia" OR "transient brain ischaemia" OR "transient cerebral ischemia" OR "transient cerebral ischaemia" OR "transient cerebral ischemic" OR "transient cerebral ischaemic" OR ("transient" AND ("brain" OR "cerebral") AND (ischemi* OR ischaem*)))) AND TI("Ethnicity" OR "Ethnology" OR "ethnic or racial aspects" OR "Ethnic Group" OR "Population Group" OR "Population Groups" OR "Population Group" OR "ethnic" OR "ethnicity" OR "ethnic*" OR "ethnology" OR "ethnol*" OR "Indians" OR "Asians" OR "Asian Americans" OR "Blacks" OR "African Americans" OR "Whites" OR "Hispanics" OR "Latino" OR "Mexican Americans" OR "Indigenous" OR "ancestry" OR "migrant" OR "undocumented immigrant" OR "Emigrants" OR "Emigrant" OR "emigrat*" OR "Immigrants" OR "Immigrant" OR "immigrat*" OR "Refugee" OR "Refugees" OR "Refugee" OR "Asylum Seekers" OR "Asylum Seeker" OR "Displaced Persons" OR "Displaced Person" OR "sociodemographic factor" OR "sociodemographic factors" OR "socio demographic factor" OR "socio demographic factors") AND (TI("Quality of Life" OR "Quality of Life" OR "QoL" OR "HRQoL" OR "life quality" OR "Sickness Impact Profile" OR "Sickness Impact Profile") OR SU("Quality of Life" OR "Quality of Life" OR "QoL" OR "HRQoL" OR "life quality" OR "Sickness Impact Profile" OR "Sickness Impact Profile") OR AB("Quality of Life" OR "Quality of Life" OR "QoL" OR "HRQoL" OR "life quality" OR "Sickness Impact Profile" OR "Sickness Impact Profile") OR MA("Quality of Life" OR "Quality of Life" OR "QoL" OR "HRQoL" OR "life quality" OR "Sickness Impact Profile" OR "Sickness Impact Profile"))))

Academic Search Premier

((TI("cerebrovascular accident" OR "Stroke" OR "strokes" OR "CVA" OR "CVAs" OR "Cerebrovascular Accident" OR "Cerebrovascular Accidents" OR "Brain Attack" OR "Brain Attacks" OR "Brain Infarction" OR "Brain Infarctions" OR "Brain Infarct*" OR "Brain Infarction" OR "Cerebrovascular Apoplexy" OR "Apoplexy" OR "Brain Vascular Accident" OR "Brain Vascular Accidents" OR "Cerebrovascular Stroke" OR "Cerebrovascular Strokes" OR "Cerebral Stroke" OR "Cerebral Strokes" OR "Acute Stroke" OR "Acute Strokes" OR "Acute Cerebrovascular Accident" OR "Acute Cerebrovascular Accidents" OR "Cerebrovascular Event" OR "Cerebrovascular Events" OR "Cerebrovascular Attack" OR "Cerebrovascular Attacks" OR "poststroke" OR "Cerebral infarction" OR "Cerebral infarctions" OR "Cerebral infarct*" OR "Brain ischemia" OR "Brain ischemia" OR "Brain ischaemia" OR "Cerebral Ischemia" OR "Cerebral Ischaemia" OR "Brain Hemorrhage" OR Cerebral Hemorrhag* OR Cerebral Haemorrhag* OR Intracerebral Hemorrhag* OR Brain Hemorrhag* OR Brain Haemorrhag* OR Intracerebral Haemorrhag* OR "Neurologic Event" OR "Neurologic Events" OR "Transient Ischemic Attack" OR "transient ischemic attack" OR "transient ischemic attack" OR "transient ischaemic attack" OR "transient ischaemic attacks" OR "TIA" OR "TIAs" OR "transient brainstem ischemia" OR "transient brainstem ischaemia" OR "transient brain stem ischemia" OR "transient brain ischemia" OR "transient brain ischaemia" OR "transient cerebral ischemia" OR "transient cerebral ischaemia" OR "transient cerebral ischemic" OR "transient cerebral ischaemic" OR ("transient" AND ("brain" OR "cerebral") AND (ischemi* OR ischaem*))) AND (TI("Ethnicity" OR "Ethnology" OR "ethnic or racial aspects" OR "Ethnic Group" OR "Population Group" OR "Population Groups" OR "Population Group" OR "ethnic" OR "ethnicity" OR "ethnic*" OR "ethnology" OR "ethnol*" OR "Indians" OR "Asians" OR "Asian Americans" OR "Blacks" OR "African Americans" OR "Whites" OR "Hispanics" OR "Latino" OR "Mexican Americans" OR "Indigenous" OR "ancestry" OR "migrant" OR "undocumented immigrant" OR "Emigrants" OR "Emigrant" OR "emigrat*" OR "Immigrants" OR "Immigrant" OR "immigrat*" OR "Refugee" OR "Refugees" OR "Refugee" OR "Asylum Seekers" OR "Asylum Seeker" OR "Displaced Persons" OR "Displaced Person" OR "sociodemographic factor" OR "sociodemographic factors" OR "socio demographic factor" OR "socio demographic factors") OR SU("Ethnicity" OR "Ethnology" OR "ethnic or racial aspects" OR "Ethnic Group" OR "Population Group" OR "Population Groups" OR "Population Group" OR "ethnic" OR "ethnicity" OR "ethnic*" OR "ethnology" OR "ethnol*" OR "Indians" OR "Asians" OR "Asian Americans" OR "Blacks" OR "African Americans" OR "Whites" OR "Hispanics" OR "Latino" OR "Mexican Americans" OR "Indigenous" OR "ancestry" OR "migrant" OR "undocumented immigrant" OR "Emigrants" OR "Emigrant" OR "emigrat*" OR "Immigrants" OR "Immigrant" OR "immigrat*" OR "Refugee" OR "Refugees" OR "Refugee" OR "Asylum Seekers" OR "Asylum Seeker" OR "Displaced Persons" OR "Displaced Person" OR "sociodemographic factor" OR "sociodemographic factors" OR "socio demographic factor" OR "socio demographic factors") OR AB("Ethnicity" OR "Ethnology" OR "ethnic or racial aspects" OR "Ethnic Group" OR "Population Group" OR "Population Groups" OR "Population Group" OR "ethnic" OR "ethnicity" OR "ethnic*" OR "ethnology" OR "ethnol*" OR "Indians" OR "Asians" OR "Asian Americans" OR "Blacks" OR "African Americans" OR "Whites" OR "Hispanics" OR "Latino" OR "Mexican Americans" OR "Indigenous" OR "ancestry" OR "migrant" OR "undocumented immigrant" OR "Emigrants" OR "Emigrant" OR "emigrat*" OR "Immigrants" OR "Immigrant" OR "immigrat*" OR "Refugee" OR "Refugees" OR "Refugee" OR "Asylum Seekers" OR "Asylum Seeker" OR "Displaced Persons" OR "Displaced Person" OR "sociodemographic factor" OR "sociodemographic factors" OR "socio demographic factor" OR "socio demographic factors") OR KW("Ethnicity" OR "Ethnology" OR "ethnic or racial aspects" OR "Ethnic Group" OR "Population Group" OR "Population Groups" OR "Population Group" OR "ethnic" OR "ethnicity" OR "ethnic*" OR "ethnology" OR "ethnol*" OR "Indians" OR "Asians" OR "Asian Americans" OR "Blacks" OR "African Americans" OR "Whites" OR "Hispanics" OR "Latino" OR "Mexican Americans" OR "Indigenous" OR "ancestry" OR

"migrant" OR "undocumented immigrant" OR "Emigrants" OR "Emigrant" OR "emigrat*" OR "Immigrants" OR "Immigrant" OR "immigrat*" OR "Refugee" OR "Refugees" OR "Refugee" OR "Asylum Seekers" OR "Asylum Seeker" OR "Displaced Persons" OR "Displaced Person" OR "sociodemographic factor" OR "sociodemographic factors" OR "socio demographic factor" OR "socio demographic factors")) AND (TI("Quality of Life" OR "Quality of Life" OR "QoL" OR "HRQoL" OR "life quality" OR "Sickness Impact Profile" OR "Sickness Impact Profile") OR SU("Quality of Life" OR "Quality of Life" OR "QoL" OR "HRQoL" OR "life quality" OR "Sickness Impact Profile" OR "Sickness Impact Profile") OR AB("Quality of Life" OR "Quality of Life" OR "QoL" OR "HRQoL" OR "life quality" OR "Sickness Impact Profile" OR "Sickness Impact Profile") OR KW("Quality of Life" OR "Quality of Life" OR "QoL" OR "HRQoL" OR "life quality" OR "Sickness Impact Profile" OR "Sickness Impact Profile"))) **OR** ((TI("cerebrovascular accident" OR "Stroke" OR "strokes" OR "CVA" OR "CVAs" OR "Cerebrovascular Accident" OR "Cerebrovascular Accidents" OR "Brain Attack" OR "Brain Attacks" OR "Brain Infarction" OR "Brain Infarctions" OR "Brain Infarct*" OR "Brain Infarction" OR "Cerebrovascular Apoplexy" OR "Apoplexy" OR "Brain Vascular Accident" OR "Brain Vascular Accidents" OR "Cerebrovascular Stroke" OR "Cerebrovascular Strokes" OR "Cerebral Stroke" OR "Cerebral Strokes" OR "Acute Stroke" OR "Acute Strokes" OR "Acute Cerebrovascular Accident" OR "Acute Cerebrovascular Accidents" OR "Cerebrovascular Event" OR "Cerebrovascular Events" OR "Cerebrovascular Attack" OR "Cerebrovascular Attacks" OR "poststroke" OR "Cerebral infarction" OR "Cerebral infarctions" OR "Cerebral infarct*" OR "Brain ischemia" OR "Brain ischemia" OR "Brain ischaemia" OR "Cerebral Ischemia" OR "Cerebral Ischaemia" OR "Brain Hemorrhage" OR Cerebral Hemorrhag* OR Cerebral Haemorrhag* OR Intracerebral Hemorrhag* OR Brain Hemorrhag* OR Brain Haemorrhag* OR Intracerebral Haemorrhag* OR "Neurologic Event" OR "Neurologic Events" OR "Transient Ischemic Attack" OR "transient ischemic attack" OR "transient ischemic attack" OR "transient ischaemic attack" OR "transient ischaemic attacks" OR "TIA" OR "TIAs" OR "transient brainstem ischemia" OR "transient brainstem ischaemia" OR "transient brain stem ischemia" OR "transient brain ischemia" OR "transient brain ischaemia" OR "transient cerebral ischemia" OR "transient cerebral ischaemia" OR "transient cerebral ischemic" OR "transient cerebral ischaemic" OR ("transient" AND ("brain" OR "cerebral") AND (ischemi* OR ischaem*))) OR SU("cerebrovascular accident" OR "Stroke" OR "strokes" OR "CVA" OR "CVAs" OR "Cerebrovascular Accident" OR "Cerebrovascular Accidents" OR "Brain Attack" OR "Brain Attacks" OR "Brain Infarction" OR "Brain Infarctions" OR "Brain Infarct*" OR "Brain Infarction" OR "Cerebrovascular Apoplexy" OR "Apoplexy" OR "Brain Vascular Accident" OR "Brain Vascular Accidents" OR "Cerebrovascular Stroke" OR "Cerebrovascular Strokes" OR "Cerebral Stroke" OR "Cerebral Strokes" OR "Acute Stroke" OR "Acute Strokes" OR "Acute Cerebrovascular Accident" OR "Acute Cerebrovascular Accidents" OR "Cerebrovascular Event" OR "Cerebrovascular Events" OR "Cerebrovascular Attack" OR "Cerebrovascular Attacks" OR "poststroke" OR "Cerebral infarction" OR "Cerebral infarctions" OR "Cerebral infarct*" OR "Brain ischemia" OR "Brain ischemia" OR "Brain ischaemia" OR "Cerebral Ischemia" OR "Cerebral Ischaemia" OR "Brain Hemorrhage" OR Cerebral Hemorrhag* OR Cerebral Haemorrhag* OR Intracerebral Hemorrhag* OR Brain Hemorrhag* OR Brain Haemorrhag* OR Intracerebral Haemorrhag* OR "Neurologic Event" OR "Neurologic Events" OR "Transient Ischemic Attack" OR "transient ischemic attack" OR "transient ischemic attack" OR "transient ischaemic attack" OR "transient ischaemic attacks" OR "TIA" OR "TIAs" OR "transient brainstem ischemia" OR "transient brainstem ischaemia" OR "transient brain stem ischemia" OR "transient brain ischemia" OR "transient brain ischaemia" OR "transient cerebral ischemia" OR "transient cerebral ischaemia" OR "transient cerebral ischemic" OR "transient cerebral ischaemic" OR ("transient" AND ("brain" OR "cerebral") AND (ischemi* OR ischaem*))) OR AB("cerebrovascular accident" OR "Stroke" OR "strokes" OR "CVA" OR "CVAs" OR "Cerebrovascular Accident" OR "Cerebrovascular Accidents" OR "Brain Attack" OR "Brain Attacks" OR "Brain Infarction" OR "Brain Infarctions" OR "Brain Infarct*" OR "Brain Infarction" OR "Cerebrovascular Apoplexy" OR "Apoplexy" OR "Brain Vascular Accident" OR "Brain Vascular Accidents" OR "Cerebrovascular Stroke" OR

"Cerebrovascular Strokes" OR "Cerebral Stroke" OR "Cerebral Strokes" OR "Acute Stroke" OR "Acute Strokes" OR "Acute Cerebrovascular Accident" OR "Acute Cerebrovascular Accidents" OR "Cerebrovascular Event" OR "Cerebrovascular Events" OR "Cerebrovascular Attack" OR "Cerebrovascular Attacks" OR "poststroke" OR "Cerebral infarction" OR "Cerebral infarctions" OR "Cerebral infarct*" OR "Brain ischemia" OR "Brain ischemia" OR "Brain ischaemia" OR "Cerebral Ischemia" OR "Cerebral Ischaemia" OR "Brain Hemorrhage" OR Cerebral Hemorrhag* OR Cerebral Haemorrhag* OR Intracerebral Hemorrhag* OR Brain Hemorrhag* OR Brain Haemorrhag* OR Intracerebral Haemorrhag* OR "Neurologic Event" OR "Neurologic Events" OR "Transient Ischemic Attack" OR "transient ischemic attack" OR "transient ischemic attack" OR "transient ischaemic attack" OR "transient ischaemic attacks" OR "TIA" OR "TIAs" OR "transient brainstem ischemia" OR "transient brainstem ischaemia" OR "transient brain stem ischemia" OR "transient brain ischemia" OR "transient brain ischaemia" OR "transient cerebral ischemia" OR "transient cerebral ischaemia" OR "transient cerebral ischemic" OR "transient cerebral ischaemic" OR ("transient" AND ("brain" OR "cerebral") AND (ischemi* OR ischaem*))) OR KW("cerebrovascular accident" OR "Stroke" OR "strokes" OR "CVA" OR "CVAs" OR "Cerebrovascular Accident" OR "Cerebrovascular Accidents" OR "Brain Attack" OR "Brain Attacks" OR "Brain Infarction" OR "Brain Infarctions" OR "Brain Infarct*" OR "Brain Infarction" OR "Cerebrovascular Apoplexy" OR "Apoplexy" OR "Brain Vascular Accident" OR "Brain Vascular Accidents" OR "Cerebrovascular Stroke" OR "Cerebrovascular Strokes" OR "Cerebral Stroke" OR "Cerebral Strokes" OR "Acute Stroke" OR "Acute Strokes" OR "Acute Cerebrovascular Accident" OR "Acute Cerebrovascular Accidents" OR "Cerebrovascular Event" OR "Cerebrovascular Events" OR "Cerebrovascular Attack" OR "Cerebrovascular Attacks" OR "poststroke" OR "Cerebral infarction" OR "Cerebral infarctions" OR "Cerebral infarct*" OR "Brain ischemia" OR "Brain ischemia" OR "Brain ischaemia" OR "Cerebral Ischemia" OR "Cerebral Ischaemia" OR "Brain Hemorrhage" OR Cerebral Hemorrhag* OR Cerebral Haemorrhag* OR Intracerebral Hemorrhag* OR Brain Hemorrhag* OR Brain Haemorrhag* OR Intracerebral Haemorrhag* OR "Neurologic Event" OR "Neurologic Events" OR "Transient Ischemic Attack" OR "transient ischemic attack" OR "transient ischemic attack" OR "transient ischaemic attack" OR "transient ischaemic attacks" OR "TIA" OR "TIAs" OR "transient brainstem ischemia" OR "transient brainstem ischaemia" OR "transient brain stem ischemia" OR "transient brain ischemia" OR "transient brain ischaemia" OR "transient cerebral ischemia" OR "transient cerebral ischaemia" OR "transient cerebral ischemic" OR "transient cerebral ischaemic" OR ("transient" AND ("brain" OR "cerebral") AND (ischemi* OR ischaem*)))) AND TI("Ethnicity" OR "Ethnology" OR "ethnic or racial aspects" OR "Ethnic Group" OR "Population Group" OR "Population Groups" OR "Population Group" OR "ethnic" OR "ethnicity" OR "ethnic*" OR "ethnology" OR "ethnol*" OR "Indians" OR "Asians" OR "Asian Americans" OR "Blacks" OR "African Americans" OR "Whites" OR "Hispanics" OR "Latino" OR "Mexican Americans" OR "Indigenous" OR "ancestry" OR "migrant" OR "undocumented immigrant" OR "Emigrants" OR "Emigrant" OR "emigrat*" OR "Immigrants" OR "Immigrant" OR "immigrat*" OR "Refugee" OR "Refugees" OR "Refugee" OR "Asylum Seekers" OR "Asylum Seeker" OR "Displaced Persons" OR "Displaced Person" OR "sociodemographic factor" OR "sociodemographic factors" OR "socio demographic factor" OR "socio demographic factors") AND (TI("Quality of Life" OR "Quality of Life" OR "QoL" OR "HRQoL" OR "life quality" OR "Sickness Impact Profile" OR "Sickness Impact Profile") OR SU("Quality of Life" OR "Quality of Life" OR "QoL" OR "HRQoL" OR "life quality" OR "Sickness Impact Profile" OR "Sickness Impact Profile") OR AB("Quality of Life" OR "Quality of Life" OR "QoL" OR "HRQoL" OR "life quality" OR "Sickness Impact Profile" OR "Sickness Impact Profile") OR KW("Quality of Life" OR "Quality of Life" OR "QoL" OR "HRQoL" OR "life quality" OR "Sickness Impact Profile" OR "Sickness Impact Profile"))))
